# Impaired mitochondrial complex I function as a candidate driver in the biological stress response and a concomitant stress-induced brain metabolic reprogramming in male mice

**DOI:** 10.1038/s41398-020-0858-y

**Published:** 2020-06-01

**Authors:** Tim L. Emmerzaal, Graeme Preston, Bram Geenen, Vivienne Verweij, Maximilian Wiesmann, Elisavet Vasileiou, Femke Grüter, Corné de Groot, Jeroen Schoorl, Renske de Veer, Monica Roelofs, Martijn Arts, Yara Hendriksen, Eva Klimars, Taraka R. Donti, Brett H. Graham, Eva Morava, Richard J. Rodenburg, Tamas Kozicz

**Affiliations:** 1 Department of Anatomy, Radboud University Medical Center, Donders Institute for Brain Cognition and Behaviour, Nijmegen, The Netherlands; 2 Department of Clinical Genomics, Mayo Clinic, Rochester, MN 55905 USA; 3 Hayward Genetics Center, Tulane University School of Medicine, New Orleans, LA 70112 USA; 4 PerkinElmer Genetics, Pittsburgh, PA 15275 USA; 5 Department of Medical and Molecular Genetics, Indiana University School of Medicine, Indianapolis, IN 46202 USA; 6 Department of Pediatrics, Radboud Center for Mitochondrial Medicine, Translational Metabolic Laboratory, Radboud University Medical Center, Nijmegen, The Netherlands

**Keywords:** Physiology, Molecular neuroscience

## Abstract

Mitochondria play a critical role in bioenergetics, enabling stress adaptation, and therefore, are central in biological stress responses and stress-related complex psychopathologies. To investigate the effect of mitochondrial dysfunction on the stress response and the impact on various biological domains linked to the pathobiology of depression, a novel mouse model was created. These mice harbor a gene trap in the first intron of the Ndufs4 gene (*Ndufs4*^GT/GT^ mice), encoding the NDUFS4 protein, a structural component of complex I (CI), the first enzyme of the mitochondrial electron transport chain. We performed a comprehensive behavioral screening with a broad range of behavioral, physiological, and endocrine markers, high-resolution ex vivo brain imaging, brain immunohistochemistry, and multi-platform targeted mass spectrometry-based metabolomics. *Ndufs4*^GT/GT^ mice presented with a 25% reduction of CI activity in the hippocampus, resulting in a relatively mild phenotype of reduced body weight, increased physical activity, decreased neurogenesis and neuroinflammation compared to WT littermates. Brain metabolite profiling revealed characteristic biosignatures discriminating *Ndufs4*^GT/GT^ from WT mice. Specifically, we observed a reversed TCA cycle flux and rewiring of amino acid metabolism in the prefrontal cortex. Next, exposing mice to chronic variable stress (a model for depression-like behavior), we found that *Ndufs4*^GT/GT^ mice showed altered stress response and coping strategies with a robust stress-associated reprogramming of amino acid metabolism. Our data suggest that impaired mitochondrial CI function is a candidate driver for altered stress reactivity and stress-induced brain metabolic reprogramming. These changes result in unique phenomic and metabolomic signatures distinguishing groups based on their mitochondrial genotype.

## Introduction

An adequate stress response comprises of various simultaneous processes which are necessary to adapt to the stressor appropriately^1^. All these processes combined require significant resources, including energy in the form of ATP, primarily synthesized by the mitochondria. Besides energy production, mitochondria are known to play a crucial role during the stress response^2^ and have even been shown to influence the stress adaptation response^3^. Furthermore, severe stress can directly influence mitochondria, primarily in a negative manner^4–7^.

As stress is one of the most important risk factors in the etiology of major depressive disorder (MDD)^8–10^, the interaction between stress and the mitochondrial function is intriguing. An increasing amount of evidence points toward the involvement of the mitochondria in the pathobiology of MDD^2,11–13^. Individuals with mitochondrial disease (MD; i.e., having severely impaired mitochondrial function) show a markedly higher prevalence of MDD compared to the general population^14,15^. Similarly, decreased mitochondrial function has been reported in MDD patients^11,12^, as well as in animal models of depression^5–7^.

Despite this accumulating evidence for mitochondrial involvement in the etiology of stress-related depression, in vivo modeling remains challenging. Animal models with mitochondrial dysfunction often show severe phenotype and are lethal at an early age, making them unsuitable for psychopathological research^16^. Here, we generated a novel mouse model with suboptimal mitochondrial function, the *Ndufs4*^GT/GT^ mice. These mice have a relatively mild phenotype in comparison to most known MD mouse models (for further details see results section). We report on a broad range of behavioral, physiological, and endocrine markers, as well as high-resolution ex vivo brain imaging, brain immunohistochemistry, and targeted mass spectrometry-based metabolomics. We hypothesized that in *Ndufs4*^GT/GT^ mice, impaired mitochondrial complex I function, would be a candidate driver event in elevated stress susceptibility and that stress-induced brain metabolic reprogramming would produce unique signatures that would discriminate stress resilience versus susceptibility.

## Materials and methods

### Generation of the novel mouse model

Mutations in the NADH dehydrogenase [ubiquinone] iron-sulfur protein 4 (*Ndufs4*) gene are associated with complex I (CI) deficiency, the most common enzymatic defect associated with oxidative phosphorylation disorders^17–20^. NDUFS4 is an 18 kDa nuclear-encoded subunit of mitochondrial CI, an essential part of the electron transport chain (ETC)^17,19,21^. Complete ablation of *Ndufs4* in mice results in a Leigh-like phenotype with multisystem involvement^21–24^. Similarly to most animal models with mitochondrial dysfunction^16^, *Ndusf4* KO mice reach humane endpoint before puberty (~7 weeks of age)^22^, making it challenging to investigate the mitochondrial etiology of affective disorders. Therefore, we sought to create a novel mouse model with lower mitochondrial function with a less severe phenotype. In order to do this, we took advantage of a gene trap insertion in the first intron of *Ndufs4*^25^ to generate a novel mouse model of a partial deficiency of the NDUFS4 protein. In short, a mouse ES cell line (129Ola) with a gene trap insertion in intron 1 of *Ndufs4* (ES cell line CB0524) was obtained from the International Gene Trap Consortium (through Sanger Institute Gene Trap Resource or SIGTR^26^). The *Ndufs4*^CB0524^ cell line was originally isolated from a large-scale gene trap experiment where 129Ola ES cells were transfected with the gene trap construct pGT0lxfT1 (^25^, https://igtc.org/). The cell line was microinjected into C57BL/6 donor blastocysts to generate transgenic line (performed by Genetically Engineered Mouse Core at Baylor College of Medicine). The mouse line used for this study was initially backcrossed for 20 generations onto the FVB/NJ inbred background. Mice used for this study were generated by heterozygous breeding.

### Chronic unpredictable stress paradigm

Chronic unpredictable stress (CUS) has been used for many years as a model for mood disorders, including depression^27,28^. We subjected 10-week-old male mice homozygous for the Ndufs4 gene trap (*Ndufs4*^*GT*/GT^) and male WT mice to CUS for 21 days with one stressor or a behavioral test each day (Fig. S1). Both WT and *Ndufs4*^GT/GT^ mice were allocated to either control or stress groups in a semi-randomized manner. This was done in a way that all groups presented similar body weights at the start of the experiment. This resulted in a total of four groups: WT control (*n* = 27), WT stress (*n* = 26), *Ndufs4*^GT/GT^ control (*n* = 26), and *Ndufs4*^GT/GT^ stress (*n* = 30). The sample size used in this study was estimated based on a power calculation following a pilot study. Twenty-four hours after the last stressor, the animals were sacrificed by cervical dislocation without anesthesia. During the stress paradigm, behavioral tests, subsequent analysis of behavioral and biochemical tests, the researchers were unaware of the mouse genotype and experimental condition at all times. All animal experiments were performed according to Dutch federal regulations for animal protection and were approved by the Central Authority for Scientific Procedures on Animals (CCD, AVD_103002016481; RU-DEC 2015-0117).

Approximately half of the animals were subjected to the forced swim test (FST), Rotarod, open field, and splash test while the other half of the animals were subjected to the tail suspension test (TST), grip test, elevated plus maze (EPM), and sucrose preference test (Fig. S1C). More detailed descriptions of all experimental procedures, including the housing of the animals, the CUS paradigm, behavioral tests, mitochondrial complex measurements, immunohistochemistry, MRI measurements, metabolomics, and statistical methods are described in SI Materials and Methods. Details on statistical outcomes are provided in Supplementary Table 1. Based on our preestablished criterion, statistically significant outliers were removed from all analysis.

### Reagents

The following antibodies were used in this study: primary polyclonal goat anti-IBA-1 (Abcam, ab-5076), polyclonal goat anti-DCX (C18) (Santa Cruz, sc-8066), and polyclonal biotinylated donkey anti-goat secondary antibody (Jackson ImmunoResearch, 705-065-003). Further details on the immunohistochemical stainings can be found in the SI Materials and Methods.

### Statistics

For statistical analysis, IBM SPSS 24 software (IBM Corporation, New York, NY, USA) was used. Five animals were removed from all analyses. Two stressed *Ndufs4*^GT/GT^ mice showed stereotypical behavior, two WT animals had a severely impaired mitochondrial CI activity whilst one *Ndufs4*^GT/GT^ animal had very high mitochondrial CI activity. To determine differences between genotype (WT or *Ndufs4*^GT/GT^) and condition (control or stressed), two-way ANOVAs were used after testing for normality using Shapiro–Wilk tests, as well as the Levene’s test for equality of error variances. When a statistically significant interaction was found between genotype and condition, the ANOVA was rerun with simple effects using a Bonferroni correction. When appropriate, repeated measures ANOVAs were performed. When only the genotype was assessed, Student’s *t*-tests were used to determine statistical significance. Detailed outcomes of the statistical tests and the sample sizes are summarized in Supplementary Table 1. Statistical significance was set at *P* < 0.05, whereas a statistical trend was set at *P* < 0.07. All data are presented as mean ± SEM.

## Results

### Ndufs4-deficiency resulted in reduced mitochondrial complex I activity

**Fig. 1:**
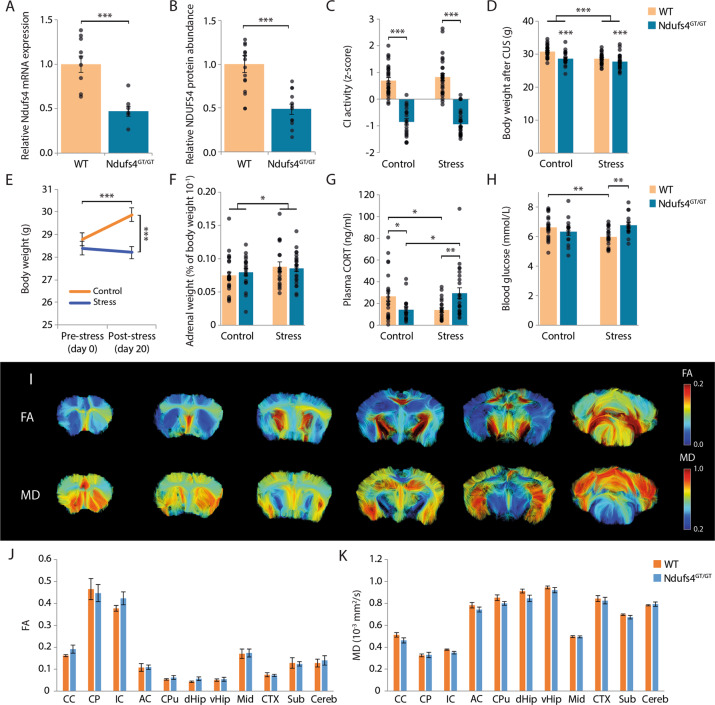
Physiological characterization of *Ndufs4*^GT/GT^ mice. **a** Relative *Ndufs4* mRNA expression normalized to *B2m* and *Gapdh* using the Pfaffl method in the brain of WT (orange bars) and *Ndufs4*^GT/GT^ (blue bars) mice. **b** NDUFS4 protein abundance relative to beta-Actin in the brain. **c***Z*-scores of mitochondrial complex I (CI) activity from hippocampal tissue. **d** Body weight as measured after 20 days of chronic unpredictable stress (CUS). **e** Body weight change from the start of chronic stress to the end of the experiment. In this graph, the genotype is not shown since there was no difference in body weight change between WT and *Ndufs4*^GT/GT^ mice over time. **f** Adrenal weight expressed as a percentage of the body weight of the animals. **g** Plasma corticosterone (CORT) levels measured one day after the last stressor. **h** Blood glucose concentrations measured with a FreeStyle Freedom Lite glucose meter using a drop of trunk blood shortly after decapitation. **i** Representative images from one animal showing the fractional anisotropy (FA) and mean diffusivity (MD) at different Bregmata. Approximately at Bregma 1.10 mm, 0.62 mm, −0.70 mm, −1.70 mm, −2.80 mm, and −6.24 mm. **j** FA was measured in several different brain regions. **k** In these same regions, MD was also measured. Graphs show the geometric mean (**a**) or average (**b**–**h, j, k**) ± SEM, with each black dot representing the result of an individual animal. *p < 0.05, **p < 0.01, and ***p < 0.001. Abbreviations: CC, corpus callosum; CP, cerebral peduncle; IC, internal capsule; AC, anterior commissure; CPu, caudate putamen; dHip, dorsal part of the hippocampus; vHip, ventral part of the hippocampus; Mid, midbrain; CTX, cortex; Sub, subthalamic regions; Cereb, cerebellum.

*Ndufs4*^GT/GT^ mice exhibited a 50% reduction in both *Ndufs4* mRNA transcript, as well as NDUFS4 protein abundance (Fig. 1a, b), resulting in a 25% reduction in CI activity in the hippocampus compared to WT littermates (Fig. 1c, Fig. S2). Mice heterozygous for the gene trap showed CI activities comparable to WTs (Fig. S2A). The activities of the other complexes of the mitochondrial respiratory chain, maximal ATP production capacity, and mitochondrial content (measured by citrate synthase (CS) activity) were not affected (Fig. S2A-B). Despite several studies showing that stress adversely affects mitochondrial function^5,7^, we found no effect of CUS on any of the ETC complexes, maximal ATP production rate, or mitochondrial content (Fig. 1c, Fig. S2B).

Individuals with *Ndufs4* mutations often present with changes in brain structure^21,24,29^. We used high-resolution *ex vivo* diffusion tensor imaging (DTI) to study brain structure in 10-week-old *Ndufs4*^GT/GT^ and WT mice (Fig. 1i). We found no differences between genotypes in various gray and white matter characteristics analyzed (Fig. 1j, k, Fig. S3A-B).

### Failure to thrive in *Ndufs4*^GT/GT^ mice

Impaired mitochondrial function often involves multiple organ systems in humans and mice, and include symptoms such as growth retardation, failure to thrive, and endocrine alterations^21–24^. We found that *Ndufs4*^GT/GT^ mice presented with failure to thrive; they were, on average, 5% lighter than their WT littermates at 13 weeks of age (Fig. 1d). Interestingly, this weight difference was not apparent at weaning age, but developed slowly over time with *Ndufs4*^GT/GT^ mice gaining less body weight each week (Fig. S4A). Animals exposed to CUS exhibited lower body weight overall, irrespective of their genotype (Fig. 1d) with an almost absent body weight increase during CUS (Fig. 1e). Mice of both genotypes showed comperable body weight change during CUS, as well as in non-stressed conditions (Fig. S4B-C). As a clear sign of chronic stress, adrenal weight was increased in both genotypes (Fig. 1f).

### Endocrine alterations in *Ndufs4*^GT/GT^ mice

Corticosterone (CORT) is a central regulator of the stress response. Comparing plasma concentrations of CORT, we found that plasma CORT was lower under baseline conditions in *Ndufs4*^GT/GT^ mice (Fig. 1g). Individuals with MD often present with diabetes^30,31^, yet, plasma glucose and insulin concentrations, as well as insulin/glucose ratio, were comparable and within physiological ranges in both genotypes (Fig. 1h, Fig. S5A-B), suggesting that *Ndufs4*^GT/GT^ mice do not present with a diabetic phenotype. However, *Ndufs4*^GT/GT^ mice exposed to CUS exhibited altered CORT and glucose dynamics. Following CUS, *Ndufs4*^GT/GT^ mice had higher plasma CORT levels, whereas WTs had lower levels compared to their non-stressed counterparts (Fig. 1g). CUS furthermore negatively affected blood glucose levels in WT mice, whereas glucose levels were not altered in *Ndufs4*^GT/GT^ mice (Fig. 1h). Despite this difference in glucose levels following CUS, no difference in plasma insulin or insulin/glucose ratio was observed (Fig. S5A-B).

### Increased physical activity of *Ndufs4*^GT/GT^ mice

**Fig. 2:**
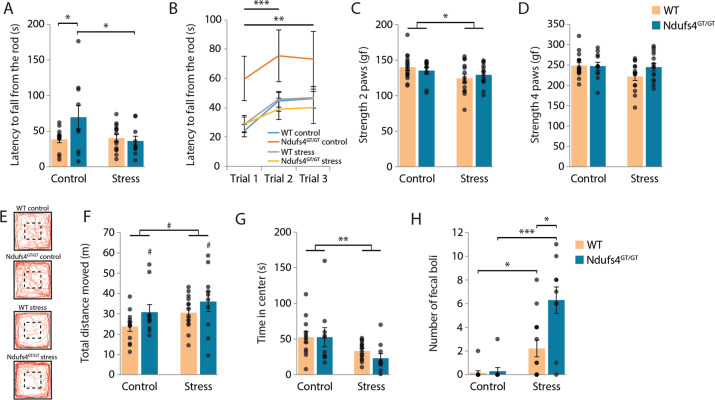
Lower mitochondrial complex I function in *Ndufs4*^GT/GT^ mice did not negatively impact locomotor function or grip strength, but *Ndufs4*^GT/GT^ mice showed slightly more anxiety-related behavior following stress. **a** Average latency to fall from a rotating rod accelerating from 4 to 40 rpm in 300s. **b** Average latency to fall from the rod during the three individual trials investigating the ability of the mice to adapt to the test. **c** The average grip strength of the animals was measured using only their forepaws or (**d**) all four paws. The grip strength test measured the peak force (in gram force, gf) of each animal. **e** Representative locomotion tracks from the open field with the center area delineated. Animals freely explored the open field for 10 min. Traces of all animals during the open field are shown in Fig. S8. **f** The total distance moved in meters, (**g**) average time spent in the center, and (**h**) defecation during the 10min in the open field were measured. Graphs show average ±SEM, with each black dot representing the result of an individual animal. *p < 0.05, **p < 0.01, and ***p < 0.001, and ^#^*p* < 0.07.

**Fig. 3:**
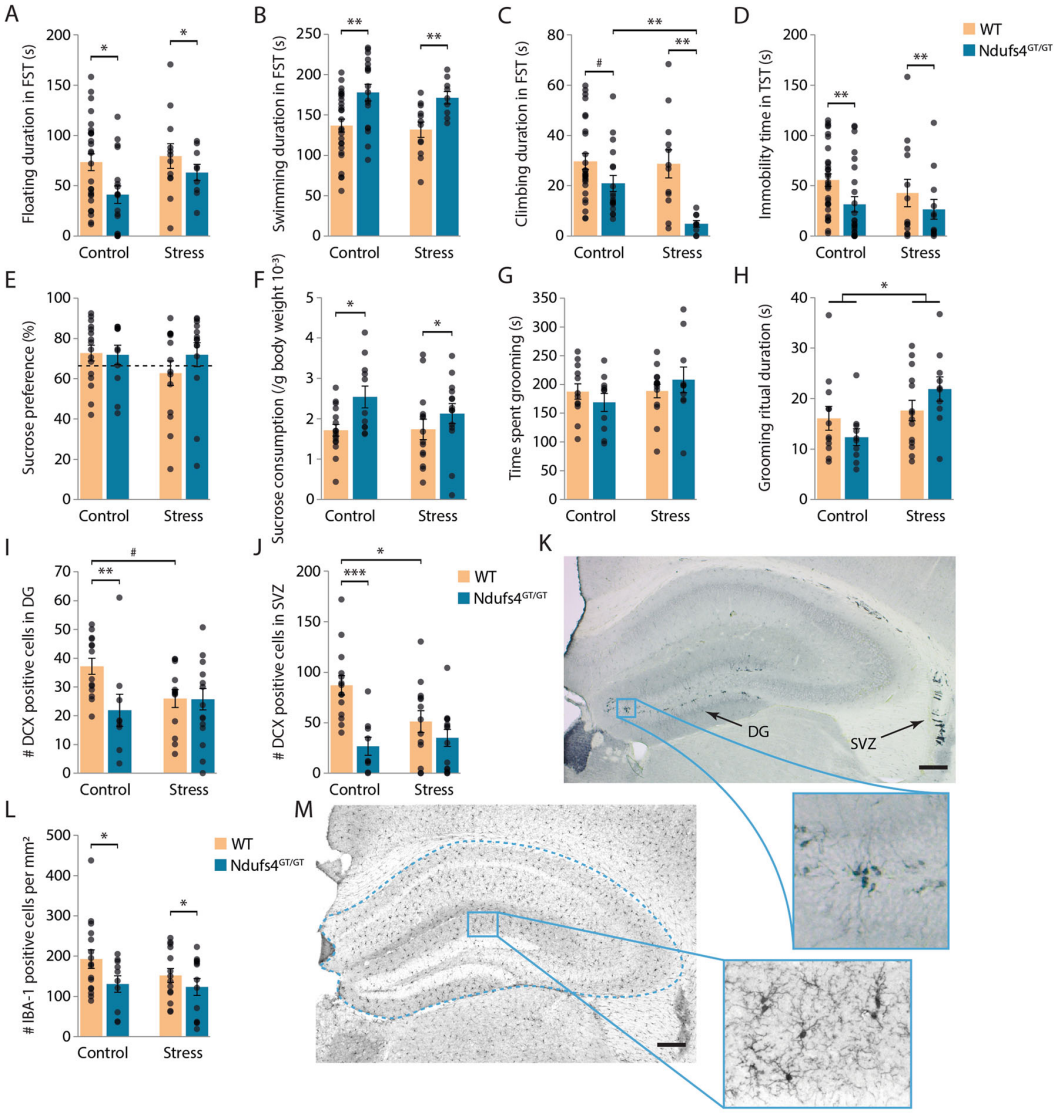
*Ndufs4*^GT/GT^ animals showed a more active coping style in the forced swim test (FST) and tail suspension test (TST), they also had less neurogenesis and inflammation in the brain. **a** The time the animals spent floating, (**b**) swimming, and (**c**) climbing was analyzed during the last 4 min of the FST. Floating was defined as minimal movement of one hind paw so the animal could stay above the water without the animal having a clear swimming direction. **d** During the TST, the total immobility time was measured. **e** During the sucrose preference test, mice could choose between two bottles, one bottle containing 1% sucrose and one bottle containing tap water. Their sucrose preference was measured as a percentage of total fluid intake. The dotted line indicates 65%, which has been suggested to be the threshold for anhedonia. **f** Total sucrose consumption was also measured. The total sucrose consumption was normalized with the body weight of each animal. **g** After spraying the dorsal coat of the animals with a 10% sucrose solution, the total time spent grooming and (**h**) average grooming ritual duration was measured. Grooming ritual was defined as the time spent grooming divided by the frequency to start grooming. **i** The number of doublecortin (DCX) positive neurons was counted as a marker for neurogenesis in the subgranular zone of the dentate gyrus (DG). **j** The number of DCX-positive neurons in the subventricular zone (SVZ) was also measured. **k** Representative image from the DCX staining showing the hippocampus with DG and SVZ (arrows). The blue rectangle represents an enlargement from the overview detailing DCX-positive cells in the DG. **l** As a proxy for inflammation in the brain, the number of Ionized calcium-binding adapter molecule 1 (IBA-1) positive microglia were analyzed in the hippocampus. The number of positive cells was normalized to the surface of the measured area. **m** Representative image from the IBA-1 staining. The number of IBA-1 cells was only counted in the hippocampus and was corrected for the hippocampal surface, as indicated by the dashed blue line. The blue rectangle represents an enlargement from the overview detailing several IBA-1 positive cells. Scale bars indicate 200 µm. Graphs show average ± SEM, with each black dot representing the result of an individual animal. *p < 0.05, **p < 0.01, ***p < 0.001, and ^#^*p* < 0.07.

MD often presents with fatigue and ataxia^21–24^, potentially influencing the performance of *Ndufs4*^GT/GT^ mice in behavioral tests. Therefore, we assessed fatigue and grip strength, motor coordination (Rotarod), and locomotion (home cage activity, open field; Fig. S1C) for potential confounding behaviors caused by the decreased complex I activity in *Ndufs4*^GT/GT^ mice. Under baseline conditions, *Ndufs4*^GT/GT^ mice showed no evidence of fatigue or muscle weakness in the Rotarod (Fig. 2a, b) and grip test (Fig. 2c, d). In the Rotarod, all mice increased their performance over time from trial 1 to trial 3 (Fig. 2b). Even in tests where high physical activity is required, such as the forced swim test (FST) and tail suspension test (TST), *Ndufs4*^GT/GT^ mice presented with no evidence of fatigue either (Fig. 3a, d). Interestingly, *Ndufs4*^GT/GT^ mice instead displayed increased physical activity in several tests when compared to WTs (Figs. 2a, f, 3a, d). *Ndufs4*^GT/GT^ mice also showed increased home cage dark phase activity under basal conditions (Fig. S6A). Following CUS, we found a similar pattern of increased physical activity of *Ndufs4*^GT/GT^ mice in the open field, FST, and TST (Figs. 2f, 3a, d). Their home cage activity showed the same pattern of increased activity following CUS; however, this did not reach statistical significance (Fig. S6B). This increased activity of *Ndufs4*^GT/GT^ mice could point to increased restlessness, a symptom often seen in individuals with MD.

### Stressed mice showed more anxiety-like behavior

Anxiety and depression are often observed as comorbid disorders^32^, and anxiety is also increasingly associated with mitochondrial dysfunction^33–35^. We measured anxiety-like behavior using the open field test or elevated plus maze (EPM, Fig. 2e, S7A, S8). Despite the increased locomotion of *Ndufs4*^GT/GT^ mice (see earlier), the time spent in the center of the open field was not different between genotypes at baseline (Fig. 2g). In the EPM, we found no difference in exploration of the apparatus (Fig. S7B-D). These data indicate that *Ndufs4*^GT/GT^ mice do not exhibit trait anxiety.

Following CUS, results from the open field suggest that animals showed more anxiety-like behavior irrespective of their genotype as stressed mice spent less time in the center of the open field (Fig. 2g). Furthermore, stress significantly increased emotional reactivity in the mice, as indicated by increased defecation during the open field test (Fig. 2h). *Ndufs4*^GT/GT^ mice exposed to CUS showed even higher emotional reactivity as they defecated more than stressed WT mice (Fig. 2h). EPM revealed no anxiety-like behavior in either genotype following CUS. Stressed mice did not show increased locomotion, nor a different exploration pattern of the EPM compared to non-stressed mice (Fig. S7B-D).

### *Ndufs4*^GT/GT^ mice showed different stress coping styles

To assess affective behavior in mice, we utilized the forced swim test (FST) and tail suspension test (TST). As previously mentioned, *Ndufs4*^GT/GT^ mice showed a more active coping strategy in the FST and TST (Fig. 3a, b, d). Interestingly, *Ndufs4*^GT/GT^ mice displayed less climbing behavior in the FST compared to WT mice (Fig. 3c). Chronic stress did not influence the floating or swimming behavior of either genotype in the FST (Fig. 3a, b). Climbing, the most energy demanding behavior in the FST, however, was influenced by CUS. *Ndufs4*^GT/GT^ mice displayed drastically reduced climbing behavior following CUS while climbing behavior in WT mice was not altered (Fig. 3c). In the TST, chronic stress did not influence behavior (Fig. 3d). Results from the FST indicate a specific coping strategy of *Ndufs4*^GT/GT^ mice; i.e., they exhibit an active, but energy-saving coping behavior.

### *Ndufs4*^GT/GT^ mice consumed more sucrose

Anhedonia is one of the core symptoms of depression. In animal models, anhedonia is assessed by the sucrose preference test^27,36^. We found no difference in sucrose preference between genotypes either under baseline conditions or following CUS (Fig. 3e). However, when calculating the amount of sucrose each animal consumes, *Ndufs4*^GT/GT^ mice consumed more sucrose (Fig. 3f).

Another symptom often observed in depression is self-neglect. This symptom can be measured using the splash test, a test to measure motivational and self-care behavior in mice. Chronic stress is often shown to decrease grooming behavior in mice^37,38^. In contrast to these studies, we found no difference in time spent grooming or duration of the grooming ritual between WT and *Ndufs4*^GT/GT^ mice (Fig. 3g–h). Also, total grooming time did not differ in either genotype following chronic stress exposure. However, the average length of a grooming ritual increased significantly in both genotypes following chronic stress (Fig. 3h).

### *Ndufs4*^GT/GT^ mice had lower neurogenesis and brain inflammation

Chronic stress is often associated with decreased adult neurogenesis^39^, and optimal mitochondrial function is necessary for adult hippocampal neurogenesis^40^. We analyzed neurogenesis in the dentate gyrus (DG) of the hippocampus and the subventricular zone (SVZ); two brain areas where adult neurogenesis is confirmed and often studied^41,42^. We used doublecortin (DCX) as a proxy to study neurogenesis (Fig. 3k). Under baseline conditions, *Ndufs4*^GT/GT^ mice showed impaired/decreased neurogenesis in both the DG and SVZ (Fig. 3i, j). Following chronic stress, WT mice exhibited decreased neurogenesis in both regions (Fig. 3i, j). However, *Ndufs4*^GT/GT^ mice did not show a further decrease in newly formed neurons (Fig. 3i, j). These results suggest that although adult neurogenesis is impaired in *Ndufs4*^GT/GT^ mice under baseline conditions, chronic stress did not further exacerbate this phenotype.

Depression is also associated with increased levels of inflammatory markers in humans^43^ and animals^44^. We quantified the number of ionized calcium-binding adapter molecule 1 (IBA-1) positive microglia in the hippocampus (Fig. 3m), a frequently used marker for inflammation in the brain. We found that *Ndufs4*^GT/GT^ mice had fewer IBA-1 positive microglia in the hippocampus (Fig. 3l). We did not find any change in the number of IBA-1 positive cells following CUS in either WT or *Ndufs4*^GT/GT^ mice (Fig. 3l).

### Decreased mitochondrial function and chronic stress resulted in metabolic rewiring

Considering the scope of metabolic perturbations in MD^45–48^ and during chronic stress^49,50^, we performed multi-platform targeted metabolomics from the prefrontal cortex focusing on amino acids (AA) and their metabolites, acyl-carnitines (AC), as well as tricarboxylic acid cycle (TCA) analytes. For a complete list of all measured metabolites, see the SI materials and methods.

#### Amino acid metabolites

**Fig. 4:**
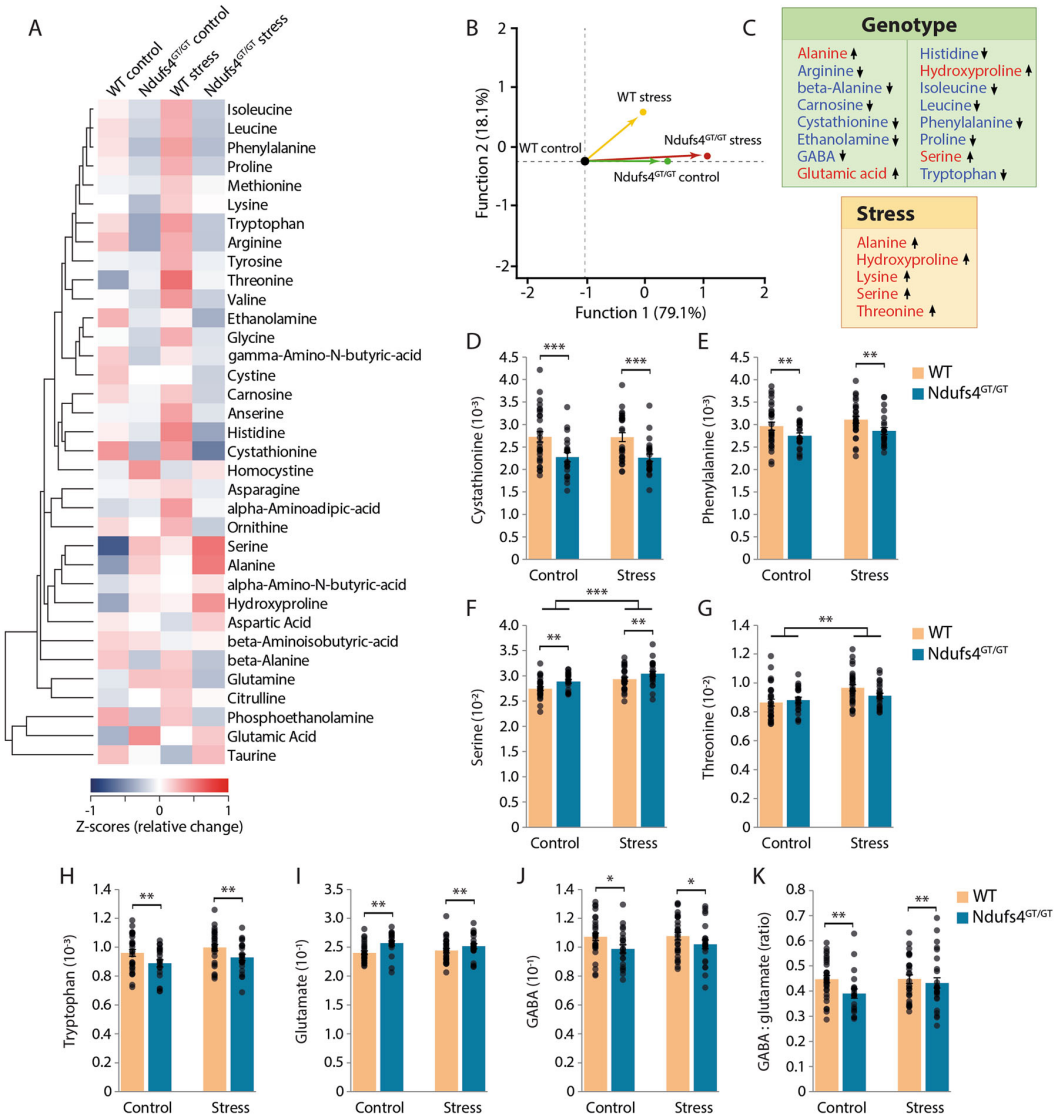
*Ndufs*^4GT/GT^ mice and chronically stressed mice show an altered metabolic rewiring. **a** Hierarchical clustering heat map of normalized (*z*-score) levels for all measured amino acid (AA) metabolites. The dendrogram indicates the degree of correlation between each of the metabolites across all four groups. **b** Stepwise linear discriminant analysis of all AA metabolites indicated that the AA profile discriminated the *Ndufs4*^GT/GT^ mice from WTs, as well as control from stress mice. **c** A highlight of the AA metabolites that were found to be statistically different between genotypes (top) or chronic stress condition (bottom). Arrows indicate the direction of the change compared to WT and control. **d**–**g** The four metabolites selected by the stepwise analysis as major contributing factors discrimination in the LDA analysis; cystathionine, phenylalanine, serine, and threonine, respectively. **h** Relative tryptophan, (**i**) glutamate, and (**j**) gamma-aminobutyric acid (GABA) levels across all four groups. **k** Relative GABA to glutamate ratio. Data were normalized to total AA concentration and show average ±SEM, with each black dot representing the result of an individual animal. *p < 0.05, **p < 0.01, and ***p < 0.001.

In preclinical studies, chronic stress-induced depression is often accompanied by abnormal AA metabolism^50,51^. Similarly, perturbations of the mitochondrial respiratory chain have also been linked to altered AA metabolism^45–48^. We found alterations of several AA metabolites in either *Ndufs4*^GT/GT^ or stressed mice (Fig. 4a, Fig. S9). Specifically, *Ndufs4*^GT/GT^ mice displayed elevated alanine concentrations in the brain. Increased alanine is often used as a clinical biomarker of mitochondrial dysfunction^52^. Besides alanine, concentrations of glutamate, hydroxyproline, and serine were increased in *Ndufs4*^GT/GT^ mice, whereas concentrations of arginine, beta-alanine, carnosine, cystathionine, ethanolamine, gamma-aminobutyric acid (GABA), isoleucine, leucine, histidine, phenylalanine, proline, and tryptophan were decreased (Fig. 4a, c, h, Fig. S9). CUS further exacerbated several of these changes; specifically, we observed a robust increase in alanine, hydroxyproline, lysine, serine, and threonine irrespective of the genotype (Fig. 4c).

Given the robust rewiring of AA metabolism in both naïve and chronically stressed *Ndufs4*^GT/GT^ mice, we next investigated whether a characteristic biosignature of AA metabolites could discriminate between experimental groups. A stepwise linear discriminant analysis (LDA) revealed that cystathionine, phenylalanine, and serine discriminated between *Ndufs4*^GT/GT^ and WT mice (Fig. 4b–f), whereas serine and threonine discriminated between stressed and non-stressed mice (Fig. 4b, f, g).

The balance between excitatory and inhibitory neurotransmission is essential for normal brain function, and deficits in both glutamate and GABA signaling have been observed in mood disorders^53–55^. We found increased glutamate and decreased GABA concentrations in the prefrontal cortex of *Ndufs4*^GT/GT^ mice (Fig. 4i, j). Consequently, the GABA to glutamate ratio was also altered (Fig. 4k), indicating a disturbed balance between inhibitory and excitatory amino acids in the prefrontal cortex.

#### Acyl-carnitine metabolites

Altered acyl-carnitines in plasma have been observed in depressed subjects^56^, as well as in individuals with MD^46^. We found that stress negatively influenced medium-chain acylcarnitines (C8) in both WT and *Ndufs4*^GT/GT^ mice (Fig. S10). There was a similar trend for C12 and C14; however, these did not reach statistical significance. Overall, lower CI activity in *Ndufs4*^GT/GT^ mice did not significantly influence acyl-carnitine metabolism (Fig. S10).

#### TCA cycle metabolites

**Fig. 5:**
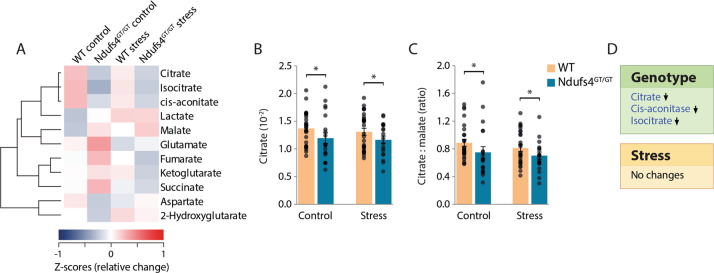
*Ndufs4*^GT/GT^ mice show signs of a reverse flux in the tricarboxylic acid (TCA) cycle. **a** Hierarchical clustering heat map of normalized (*z*-score) levels for all measured TCA cycle metabolites. The dendrogram indicates the degree of correlation between each of the metabolites across all four groups. **b** Relative citrate levels measured from the prefrontal cortex. **c** Relative citrate to malate ratio. **d** A highlight of the TCA metabolites that were found to be statistically different between genotypes (top) or chronic stress condition (bottom). Data were normalized to total TCA concentration and show average ± SEM, with each black dot representing the result of an individual animal. Asterisk (*) indicates *p* < 0.05.

Besides alterations in AA metabolism, individuals with MD and in vivo models also show alterations in TCA cycle metabolism^46,47^. We also identified disruptions in TCA cycle metabolism in *Ndufs4*^GT/GT^ mice (Fig. 5a–d). More specifically, we found that *Ndufs4*^GT/GT^ mice had a lower concentration of citrate (Fig. 5b), cis-aconitate, and isocitrate (Fig. S11). The combination of decreased citrate and isocitrate, the unaltered abundance of α-ketoglutarate, but the decreased ratio of citrate to malate (Fig. 5c) could be indicative of reverse flux in the TCA cycle in *Ndufs4*^GT/GT^ mice. Besides these genotypic alterations, chronic stress did not influence TCA metabolism (Fig. 5d, Fig. S11).

## Discussion

To test the hypothesis that suboptimal mitochondrial function increases susceptibility to stress-related psychopathologies, such as depression, we used a novel mouse model with a partial deficiency of mitochondrial complex I function due to lower NDUFS4 protein abundance (*Ndufs4*^GT/GT^ mice). In contrast to the severe phenotype of the *Ndufs4* KO mouse^22^, *Ndufs4*^GT/GT^ mice only presented with a mild phenotype of failure to thrive, hyperactivity, and increased sucrose consumption. In addition to this, we found that *Ndufs4*^GT/GT^ mice had altered plasma CORT and glucose dynamics, as well as lower adult neurogenesis and brain inflammation. Despite the lower adult neurogenesis, we found no changes in the brain structure of *Ndufs4*^GT/GT^ mice using ex vivo high-resolution DTI. These findings indicate that a 25%-30% decrease in CI activity is not sufficient to cause comparable (severe) physiological, endocrine, and behavioral symptoms to those seen in *Ndufs4* KO mice between 5 and 7 weeks of age and individuals with Leigh syndrome^22,29^.

We found no effect of the decreased CI activity on the maximal ATP production rate in the hippocampus. Although this might be surprising, it is in line with the prediction by Alam, et al.^57^ suggesting that mouse skeletal muscle mitochondria possess a substantial CI overcapacity, which minimizes the effects of CI dysfunction on mitochondrial metabolism. In one of their computational models, they predicted that cellular ATP levels would only drop after a 90% reduction in CI activity^57^. Similar lack of change in cellular ATP level has been shown in other animal and cellular Ndufs4 models, which show no, or only marginally decreased ATP production while CI activity is severely decreased^22,57–60^. In addition, there may be several other compensatory mechanisms to maintain cellular ATP levels upon decreased CI activity, such as increased activities of other ETC complexes, or increased TCA cycle activity^22,57^.

Taken together, the fact that *Ndufs4*^GT/GT^ mice only exhibit a mild phenotype under basal unstressed conditions makes them a valuable animal model for studying complex disorders associated with suboptimal mitochondrial function^2,61^ that require behavioral assessments, albeit less suitable for studying mitochondrial disease due to complex I dysfunction (Leigh syndrome).

To investigate the involvement of mitochondrial function in complex stress-associated disorders such as depression and anxiety, we subjected *Ndufs4*^GT/GT^ mice to a chronic unpredictable stress (CUS) paradigm; a model frequently used to induce depression/anxiety-like phenotype in rodents^28^. We observed that the CUS paradigm was successful in causing significant distress in both WT and *Ndufs4*^GT/GT^ mice, as indicated by an attenuated body weight gain, an increased relative adrenal weight, and increased open field locomotion. In addition to these findings, *Ndufs4*^GT/GT^ mice presented with altered CORT and glucose dynamics following stress, potentially making them more vulnerable to stress-associated disorders, such as diabetes. This altered CORT response following CUS is in line with a previous study showing distinct CORT responses in various animal models with different mitochondrial dysfunctions after acute restraint stress^3^.

We could not, however, replicate findings that stress directly influenced mitochondrial function^4–7^. One possibility may be the time point chosen to analyze stress-induced changes in mitochondrial markers; i.e., all mice in our study were sacrificed 24 h after the last stressor, whereas in studies where stress-related mitochondrial dysfunction has been reported, mice were sacrificed immediately after the last stressor or did not specify the timeframe^4–7^. Another possibility for this may be the fact that we measured ETC complex activities in the hippocampus. We chose the hippocampus to measure ETC complex activities because it has been implicated in the etiology of depression^62^. However, based on our relatively large set of unpublished data on ETC complex activities in the hippocampus, and the fact that changes in mitochondrial function have been limited to the cortex and cerebellum^5–7^, a picture is emerging that the hippocampus seems rather resilient to mitochondrial dysfunction. Clinical imaging data from individuals with MD also supports this: gray matter lesions are often found in the cortex, thalamus, brainstem, and basal ganglia^21,24,29^ while the hippocampus seems to be preserved (personal communication to Amy Goldstein, Center for Mitochondrial Medicine, CHOP Philadelphia).

Besides these physiological differences following stress, we also demonstrated that *Ndufs4*^GT/GT^ mice presented with altered stress coping strategies in the FST. Specifically, *Ndufs4*^GT/GT^ mice prefer an active but less energy-consuming coping strategy. This difference from WT mice was already apparent at baseline but was further exacerbated by chronic stress. *Ndufs4*^GT/GT^ mice showed a similar coping strategy in the TST too. Furthermore, we found that *Ndufs4*^GT/GT^ mice presented with increased emotionality following CUS as indicated by the increased defecation in the open field^63–65^

Depression has frequently been associated with decreased neurogenesis^39^. In support of this, we also found reduced neurogenesis in WT mice exposed to CUS. In agreement with earlier studies^40,66^, we also demonstrated that suboptimal mitochondrial function negatively influences neurogenesis in *Ndufs4*^GT/GT^ mice. The decreased neurogenesis in *Ndufs4*^GT/GT^ mice at baseline could be mediated by the decreased number of microglia we found in the brain, as they also play a positive role during adult neurogenesis^67^. This difference in baseline neurogenesis could make *Ndufs4*^GT/GT^ mice more vulnerable to stress as it has been shown that ablation of neurogenesis can enhance stress susceptibility^68^. The combination of chronic stress and decreased mitochondrial function did not further impair neurogenesis, probably because of a potential floor-effect as a result of the already low number of newly made neurons in *Ndufs4*^GT/GT^ mice.

Taken together, *Ndufs4*^GT/GT^ mice seem to recapitulate several phenotypes of the High Reactivity (HR) mice; a mouse model postulated to model the melancholic depressive behavior^69^. Similar to HR mice, our *Ndufs4*^GT/GT^ mice show hyperactivity and hyperactive coping strategies in most behavioral tests, decreased body weight, decreased neurogenesis, and altered CORT reactivity.

Using multi-platform targeted metabolomics, we also found abnormal amino acid metabolism, an indication towards a reverse flux in the TCA cycle, but no specific perturbation in acyl-carnitine metabolites following chronic stress. The profound alterations in AA biosignatures in both control and stressed *Ndufs4*^GT/GT^ mice point to a metabolic rewiring, as defined by the change in mitochondrial activity and subsequent alteration/redistribution of intracellular energy flow and metabolism, driven by altered brain energy metabolism. These biosignatures largely overlap with previous clinical and preclinical studies of mitochondrial dysfunction^45–47^, indicating that alterations in AA metabolites could be a characteristic biosignature for altered energy flow in the brain. If one focusses on specific AA metabolites, the elevation in alanine concentration in *Ndufs4*^GT/GT^ mice is particularly noteworthy. Alanine is often elevated in clinical and preclinical models of MD and therefore considered a biomarker for MD^45^.

Four amino acids—cystathionine, phenylalanine, serine, and threonine—were identified by a stepwise LDA to be able to discriminate between genotypes and stress conditions. Serine and cystathionine have frequently been found to be elevated in MD patients^70–73^. In agreement with previous studies, we found elevated serine in *Ndufs4*^GT/GT^ mice; however, cystathionine was decreased in the brains of *Ndufs4*^GT/GT^ mice. This latter finding is unusual since most studies show an elevated cystathionine in MD^70–73^. This discrepancy could be because we measured the amino acids in the brain, whereas other studies usually assay other tissues or plasma. Also, within the brain, different regions may show different amino acid alterations following stress exposure^74,75^. Taken together, these findings indicate alterations in the one-carbon metabolism in *Ndufs4*^GT/GT^ mice, probably supporting increased glutathione synthesis, a potent antioxidant^76^.

Besides the parallel alterations in AA metabolites in our animal model and previously published literature, we also identified distinct changes in AA metabolites. These could point to (1) particular biosignatures for a specific clinical or preclinical model of mitochondrial dysfunction;^45,48^ (2) biosignatures that differentiate based on the severity of mitochondrial dysfunction^77^; (3) specific alterations for the brain region we analyzed.

Several studies demonstrate that chronic stress exposure, depression, and anxiety cause alterations in both excitatory glutamate, as well as inhibitory GABA neurotransmission^54,74,75^. Similar to these studies, we observed increased glutamate and decreased GABA levels in the PFC of *Ndufs4*^GT/GT^ mice, resulting in an altered balance between these inhibitory and excitatory neurotransmitters. Several human studies reported lower mPFC GABA levels in MDD, and reduced mPFC GABA levels have been associated with treatment resistance^78^. These findings suggest that GABA may play a crucial role in multiple pathways leading to mPFC structural deficits in depression^54,78^. In addition, our finding of increased emotional reactivity of *Ndufs4*^GT/GT^ mice is in line with a recent review suggesting that mitochondria could contribute to anxiety through alterations in the GABA/glutamate ratio^79^.

Another neurotransmitter of interest could be serotoninergic neurotransmission. Although we did not measure serotonin levels directly, we found decreased levels of tryptophan in *Ndufs4*^GT/GT^ mice. Tryptophan metabolism is essential in the synthesis of serotonin since tryptophan is the sole precursor for serotonin. This finding is in line with previous studies that observed decreased tryptophan levels in depressed patients^80,81^. In turn, this could indicate a shunted metabolism of tryptophan away from serotonin production and increased synthesis of detrimental tryptophan catabolites^80,82,83^.

The results of this study have to be considered with some limitations. First, the *Ndufs4*^GT/GT^ mice analyzed here were in an FVB background. This could attribute to the lack of classical behavioral changes following CUS in both WT and *Ndufs4*^GT/GT^ mice, despite the several physiological, biochemical, and histological changes found in this study. It is not uncommon for FVB mice to show behavioral resilience following chronic stress^84^, and FVB mice usually show low inactive behaviors in the FST and TST^85,86^. Apparently, the physiological, biochemical, and histological changes induced by stress do not correlate to the behavior in this mouse strain following chronic stress. Second, mice with the FVB background harbor a mutation in the ATP8 mtDNA gene^87^. Mutations in this gene have been, in addition to the ATP6 gene, implicated in individuals with a family history of psychiatric disorders^88^. Therefore, when comparing data from FVB mice (this study) and those of the literature, which mostly investigate C57BL/6J mice, a potential strain difference has to be taken into consideration. Future studies are warranted to address this potential limitation. Third, all mice had to be individually housed, as individual housing is recommended by the Institute for Laboratory Animal Research for FVB mice^89^ due to the highly aggressive nature of this strain, even towards littermates^90,91^.

We conclude that stress-induced behavioral, endocrine, metabolic, and physiological responses merged into unique signatures that distinguished groups based on their mitochondrial genotype. In addition, we confirmed a link between impaired brain energy metabolism and altered biological stress response of increased emotionality, hyperactivity and hyperactive coping strategies, decreased body weight and neurogenesis, as well as altered CORT reactivity resembling a melancholic depression phenome. Upon interpretation of these results, one should, however, consider that the biological stress response, as well as the regulation of mood, are comprised of multiple endocrine, physiological, and behavioral domains. Thus, this novel *Ndufs4*^GT/GT^ mouse could be a valuable in vivo model system to study specific biological domains/endophenotypes of depression and anxiety that could be more vulnerable to impaired brain bioenergetics. Our model could also serve as a platform for identifying novel therapeutic avenues for complex psychopathologies associated with suboptimal mitochondrial function.

## Supplementary information

Supplementary information

Figure S1

Figure S2

Figure S3

Figure S4

Figure S5

Figure S6

Figure S7

Figure S8

Figure S9

Figure S10

Figure S11

Table S1
