# Supplementary material for: Impaired mitochondrial complex I function as a candidate driver in the biological stress response and a concomitant stress-induced brain metabolic reprogramming in male mice
Source: Transl Psychiatry. 2020 Jun 1;10:176. doi: 10.1038/s41398-020-0858-y (PMC7266820; doi:10.1038/s41398-020-0858-y)
Supplement: Supplementary file 1 — Supplementary information [file 41398_2020_858_MOESM1_ESM.docx]

Supplementary Information for

***Impaired mitochondrial complex I function as a candidate driver in the biological stress response and a concomitant stress-induced brain metabolic reprogramming in male mice***

Tim L. Emmerzaal, Graeme Preston, Bram Geenen, Vivienne Verweij, Maximilian Wiesmann, Elisavet Vasileiou, Femke Grüter, Corné de Groot, Jeroen Schoorl, Renske de Veer, Monica Roelofs, Martijn Arts, Yara Hendriksen, Eva Klimars, Taraka R. Donti, Brett H. Graham, Eva Morava, Richard J. Rodenburg, Tamas Kozicz

Corresponding author: Tamas Kozicz

Email: Kozicz.Tamas@mayo.edu

**This file includes:**

Supplementary Material and Methods

References for SI reference citations

Supplementary Figures S1 to S10

**Other supplementary materials for this manuscript include the following:**

Supplementary Table 1. Summary of statistics

# Supplementary Materials and Methods

## Animals

The *Ndufs4* deficient mouse model (*Ndufs4*^GT/GT^ mice) used in the current study was a generous gift from Dr. Brett H. Graham at the Department of Molecular and Human Genetics, Baylor College of Medicine, Houston, TX, USA. These animals were created by a gene trap insertion in the first intron of the *Ndufs4* gene resulting in a decreased NDUFS4 protein. This gene trap insertion results in a 25% lower mitochondrial complex I activity in the hippocampus compared to WT animals. Founder *Ndufs4*^GT/GT^ animals were on an FVB/NJ background. A colony was started at the central breeding facility of the Radboud university medical center, The Netherlands.

Mice were genotyped with a gene-specific common forward primer (AACAGAGGTGGAGTAATGCAGTCA) for wild-type and mutants, and a gene-specific reverse primer (TCCTCTGACCTTGGTACAAGTTCAC) for wild type and gene trap specific reverse primer (CTTTCTCTGTGCAAGAAGCATGAAT) for mutants. PCR product was subjected to agarose gel electrophoresis, and the wild-type and mutant bands were identified based on the size (Wt-1199bp and Mutant-880bp).

For the current study, WT and *Ndufs4*^GT/GT^ mice were generated by heterozygous breeding. A total of 106 male mice were used. Two weeks before the start of the experiments, animals were housed individually and transported to the central animal facility at the Radboud university medical center, Nijmegen, The Netherlands. Here, all experiments took place in the Preclinical Imaging Centre (PRIME) between 8 a.m. and 5 p.m. Solitary housing was used to prevent abnormal behavior caused by fighting or suppression by the alpha male. The FVB strain is known for its aggressive nature, even towards littermates ^1^. At the start of the experiment, animals had an average age of 9.8 weeks (8.14-11.57 weeks) weighing 27.4 gram (21.3-33.3 g).

The animals were kept in standard digital ventilated cages (DVC; GM500 model, Techniplast S.p.A, Buggugiate, Italy) with *ad libitum* access to food (R/M-H maintenance, ssniff®) and tap water. The cage floor was covered with corn cobs bedding material, cardboard nesting material, and a plastic shelter. Room temperature was kept at a constant 21 °C with a relative humidity of 50-60% and an artificial 12:12 light-dark cycle (lights on at 7 a.m.). The experiments were performed according to Dutch federal regulations for animal protection and were approved by the Central Authority for Scientific Procedures on Animals (CCD, AVD_103002016481; RU-DEC 2015-0117).

## Chronic unpredictable stress paradigm

Ten days before the start of the stress paradigm, animals arrived at the animal facility to acclimatize. During the whole study, all animals were weighed every five days to follow body weight changes (**Fig. S1A**). Both WT and *Ndufs4*^GT/GT^ mice were allocated to two groups in a semi-randomized manner in a way all groups presented similar body weights. This resulted in a total of four groups: WT control (n=27), WT stress (n=26), *Ndufs4*^GT/GT^ control (n=26), and *Ndufs4*^GT/GT^ stress (n=30). Animals in the stress groups were subjected to chronic unpredictable stress (CUS) consisting of different physical and psychological stressors as well as different behavioral tests (**Fig. S1B, C**). Animals in the control groups only received the behavioral tests on the same days as the stressed animals. Approximately half of the animals were subjected to the forced swim test (FST), Rotarod, open field, and splash test while the other half of the animals were subjected to the tail suspension test (TST), grip test, elevated plus maze (EPM), and sucrose preference test (**Fig. S1C**). Stressors or behavioral tests were presented once daily in a semi-randomized order for 21 days. Stressors included social housing (3x 30 min), restraint (30 min), overnight damp bedding material, shaking stress (60 min), 45° tilted cage (6 h), exposure to 4 °C (60 min), overnight food deprivation, and exposure to a novel environment (24 h). All stressors were presented twice, except for restraint, which was presented three times (**Fig. S1B**). During the stress paradigm, the researchers were unaware of the mouse genotypes at all times.

## Behavioral tests

During all behavioral tests, the observer was unaware of the genotypes and treatment conditions. All behavioral tests were performed in a dimly illuminated room after the animals had been habituated for minimally 30 minutes. Behavioral tests that were recorded were analyzed at a later time point with EthoVision XT12 (Noldus, Wageningen, the Netherlands). Also, the person that scored the videos was blind to genotypes and treatment conditions at all times.

### Physical performance tests

The Rotarod or grip strength test was used to assess if lower mitochondrial function or stress had an effect on motor function and strength. To habituate the mice to the Rotarod apparatus (IITC Life Science, Los Angeles, USA), they were placed on the stationary rod for minimally one minute, after which a test trial was started. In total, three more trials were executed with a minimum inter-trial interval of 20 minutes. During all trials, the rod turning speed accelerated from 4 to 40 rounds per minute (rpm) in 300 seconds. From each trial, the latency to fall from the rod was recorded as an outcome measure. For data analysis, the best performance of the mice from the three trials was used.

The strength of the mice was measured with a grip strength meter (Grip-Strength Meter, 47200, Ugo Basile, Italy). Animals either had to grab a trapeze with their forepaws or a grid with all four paws, after which they were pulled backward inducing a pulling behavior of the mice. A total of 6 consecutive trials for both the trapeze and grid were used with a minimum of 60 minutes between trapeze and grid measurements. The recorded peak force (in gram force; gf) was used as an outcome measure. For data analysis, the best two trials were averaged to obtain a single measurement per mouse.

### Anxiety-related tests

An open field or an elevated plus maze (EPM) was used to investigate the effect of a lower mitochondrial function and stress on general locomotor activity, exploratory behavior, and anxiety-related behaviors. On day 19 of the chronic unpredictable stress paradigm, the mice were either placed in the center of a 45x45(x30) cm clear Plexiglas arena (open field) or an EPM. The behavior of the mice was recorded for 10 minutes using EthoVision XT12 in a top-down view for both tests. The arena of the open field was divided into different zones, including the center. Distance traveled and time spent in each of the predefined areas was recorded. After each trial, the number of fecal boli the animals left behind was also noted.

The EPM apparatus consisted of two open and two closed arms elevated 60 cm from the floor. Both open and closed arms extended 35 cm from the center, which was 10x10 cm. Time spent and distance traveled in each of the arms was recorded and analyzed.

### Behavioral despair related tests

To determine behavioral despair in WT and *Ndufs4*^GT/GT^ mice before and after stress, a forced swim test (FST) or a tail suspension test (TST) was used. Both tests were executed twice, the first time at the start of the CUS paradigm and the second time at day 20 of the CUS. The first measurement included all animals in determining their baseline behavior. For the second measurement, only stressed mice were subjected to either the FST or TST.

The FST was executed by placing a mouse in a glass beaker (18x13 cm) filled with 1.8L water. The water level was high enough that the animals were not able to touch the bottom of the glass with their tails and had a temperature between 22-23 °C. A digital camera was used to record the behavior of the animals in a frontal view for 6 minutes. After each trial, the beaker was cleaned, and fresh water was used for each new animal. Only the last 4 minutes of each trial were used to score the following behaviors in EthoVision XT12: climbing, swimming, and floating. Floating was defined as the minimal movement of hind paws so the animal could stay above the water without the animal having a clear swimming direction.

For the TST, the animals were suspended by their tail at a height of approximately 40 cm using tape. The tail was covered with a hollow cylinder of 4x1.6cm to prevent the animals from tail climbing. The behavior of the animals was recorded in a similar fashion to the FST for 6 minutes. The time the animals were immobile was used as an outcome measure, analyzed using EthoVision XT12.

### Anhedonia related tests

At day 21 of the CUS paradigm, animals either underwent a splash test or a sucrose preference test. The splash test was used to indicate self-care and motivational behavior. In this test, a 10% sucrose solution was sprayed on the dorsal coat of the animal to induce grooming behavior. After spraying the 10% sucrose on their coat, the animals were placed back in their home cage without cage enrichment. Here, their behavior was recorded from a side view with a digital camera for 5 minutes. The grooming duration and frequency of grooming initiation were scored. From these two parameters, the duration of the grooming ritual was calculated.

The sucrose preference test was used to indicate anhedonic behavior in the animals. Three days before the sucrose preference test, two water bottles were placed in the home cage of the animal; both filled with water. Each day the weight of the bottles was measured to determine their drinking behavior and if the animals had any preference towards a specific bottle. On day 21 of the stress paradigm, one of the bottles was filled with a 1% sucrose solution. After 24 hours, both bottles were weighed again, and the total fluid consumption of the two bottles was calculated. Sucrose preference as a percentage of the total fluid consumption, as well as total sucrose consumption per gram of body weight, were used as outcome measurements. No food or water deprivation was applied before this test.

### Day-night rhythm

Because the animals were kept individually in DVC cages, their day-night rhythm could easily be investigated. The DVC cages contained 12 sensors to monitor the home cage activity of the animals during the whole experiment ^2^.

## Tissue processing

One day after the last behavioral test, the animals were sacrificed by cervical dislocation, without anesthesia, followed by decapitation. A drop of blood was used to measure glucose concentration with a FreeStyle Freedom Lite glucose meter (Abbott Diabetes Care Ltd.). The rest of the trunk blood was collected in 0.5 ml MiniCollect K3E K3EDTA tubes (Greiner Bio-One). After shaking shortly, the tubes were put on ice until centrifuged at 3000 g for 10 minutes at 4 °C. The supernatant (plasma) was transferred to a new tube, snap-frozen in liquid nitrogen, and stored at -80 °C until further use. Furthermore, adrenals and the brain were collected. All adrenals were snap-frozen in liquid nitrogen and stored at -80 °C until further use. From the animals that were subjected to the FST, Rotarod, open field, and splash test, the left hemisphere was snap-frozen in liquid nitrogen while the right hemisphere was immersion fixated in 4% formaldehyde (phosphate-buffered) at 4 °C for approximately 20 hours. From the animals that were subjected to the TST, grip test, EPM, and sucrose preference test, the whole brain was snap-frozen. New randomized numbers were given to the collected biological material.

### Adrenal weight

The adrenal weight was measured as an indirect measurement of the activity of the adrenal glands. Before weighing the adrenals on a precision scale, all the surrounding fatty tissue was removed. The adrenal weight was normalized to the body weight of each mouse to obtain a relative weight.

## Mitochondrial complex measurements

The left hippocampi were dissected and used to measure the individual complexes of the respiratory chain, succinate cytochrome c oxidoreductase (SCC), citrate synthase (CS) enzyme activities, and ATP production rates. A crude homogenate of fresh hippocampus tissue was made with a Teflon pestle in SEF buffer (0.25M sucrose, 2 mM EDTA in 10 mM kPi, pH 7.4) to obtain a homogenate of approximately 5%. Following homogenization, the homogenate was centrifuged at 600g for 10 minutes at 2 °C. The 600g supernatant was aliquoted in 100 µl samples, snap-frozen in liquid nitrogen and stored at -80 °C for following mitochondrial complex measurements. All procedures were carried out on ice.

All enzyme activities were measured spectrophotometrically on a KoneLab 20XT analyzer (Thermo Scientific) following standard procedures ^3^. The enzyme assays are based on previously described methods ^4-8^ and depend on intact, active enzyme complexes to catalyze the conversion of the specific substrates. These conversions can be measured spectrophotometrically, after which the data was analyzed, and the activity of the individual complex was calculated. To expose the OXPHOS complexes for enzyme activity assessment, the 600g supernatant underwent 3 freeze-thaw cycles to permeabilize the mitochondria adequately.

### Mitochondrial complex I

Mitochondrial complex I (CI) activity was measured according to previously described methods ^5^. The terminal electron acceptor used in this method was 2,6-dichlorophenolindophenol (DCIP). In short, CI oxidizes NADH; the produced electrons reduce the substrate coenzyme Q1, which subsequently reduces DCIP. The reduction of DCIP can be followed spectrophotometrically at 600nm. Rotenone, a specific inhibitor of complex I, was added as a blank measurement.

### Mitochondrial complex II

For determining the complex II (CII) activity, DCIP was also used as the terminal electron acceptor, as previously described ^8^. In short, CII converts succinate + FAD to fumarate + FADH_2_. Electrons from FADH_2_ are subsequently transferred to DUB that forms DUBH and reduces DCIP. This reduction of DCIP can again be followed at 600nm. Malonate, a specific inhibitor of CII, was added as a blank measurement.

### Mitochondrial complex III

The activity of complex III (CIII) was measured with the artificial substrate decylubiquinone-H_2_ (DUH_2_) and cytochrome C, which is converted by CIII into reduced decylubiquinone and subsequently reduced cytochrome C ^6^. This conversion results in an increased extinction at 550nm. The formed reduced cytochrome C cannot be oxidized by cytochrome C-oxidase (COX) because this enzyme was inhibited by the addition of NaN_3_ to the reaction mixture.

### Mitochondrial complex IV

The activity of complex IV was measured by determining the activity of cytochrome C oxidase (COX) ^4^. COX oxidizes reduced cytochrome C, in which oxygen is reduced to water. The decrease of reduced cytochrome C can be measured at 550nm.

### Mitochondrial SCC activity

We also measured the activity of succinate cytochrome c oxidoreductase (SCC). This assay investigates the CII -> CoQ_10_ -> CIII route. An alteration in SCC activity can point to an alteration of CII or CIII activity or a change in CoQ_10_ levels. The SCC enzyme activity was determined by measuring the reduction of cytochrome C with succinate as a substrate. To eliminate the reduction of cytochrome C via electrons coming from complex I, rotenone was added. To prevent the oxidation of reduced cytochrome C via complex IV, NaN_3_ was added. An increased extinction at 550nm of reduced cytochrome C is a measure for the SCC activity.

### Citrate synthase activity

The above analyses give insight into the activity of the individual complexes. However, when more mitochondria are present in one sample compared to other samples, the absolute activity of the complexes will also be higher because of the increased number of complexes. As citrate synthase (CS) is a measure of the number of mitochondria, all measurements were normalized to CS. Citrate synthase is a matrix enzyme of the TCA/Krebs cycle that catalyzes the conversion of oxaloacetate and acetyl-CoA to citrate and CoA. Besides the substrates acetyl-CoA and oxaloacetate, DTNB (5',5'dithiobis-(2nitrobenzoaat)) was added, which forms a complex with CoA that has an absorption spectrum at 412nm. An increased absorption at this wavelength is a measure of CS activity. As a blank measurement, the absorption at 412nm was measured without the substrate oxaloacetate ^7^.

### ATP and CrP analysis

To determine the maximal ATP and phosphocreatine (CrP) production rate, freshly prepared 600g supernatant was used according to previously described methods ^9^. The supernatant was incubated in two incubation media for 20 minutes at 37 °C. One buffer contained 30 mM K+-phosphate buffer, 75 mM KCl, 8 mM Tris, 1.3 mM K-EDTA, 0.2 mM P1,P5-Di (adenosine-5′) pentaphosphate (Ap5A), 0.5 mM MgCl_2_, 2 mM ADP, 20 mM creatine, 1 mM malate, and 1 mM pyruvate. The second incubation medium was the same but with 2 mM arsenite blocking residual glycolysis. The reaction was stopped after 20 minutes with HClO_4_ and putting the sample on ice. After the samples were centrifuged, 100 µl supernatant was neutralized with 120 µl 0.333 M KHCO_3_ and 1:1 diluted with MQ. The samples were stored at -20 °C until analysis on the KoneLab.

## NDUFS4 protein abundance

Intact brain tissue was lysed in RIPA Lysis and Extraction Buffer (Thermo Fisher Scientific 89900) with a 1% Phosphatase Inhibitor Cocktail (Thermo Fisher Scientific) and 1% Protease Inhibitor Cocktail (Thermo Scientific). Tissues were initially disrupted with a tissue pestle, before being aspirated several times through a 22G hypodermic needle. Lysates were centrifuged at 14,000 rpm for 10 minutes at 4 °C, and the supernatant was collected and stored at -80 °C. The protein concentration of the supernatant was determined using the Pierce BCA Protein Assay Kit (Thermo Scientific).

15 µg isolated protein was separated on a 12% bis-tris protein gel and transferred to a nitrocellulose or PVDF membrane according to the Invitrogen Bis-Tris Western Blotting Protocol. Membranes were blocked in SEA Block Blocking Buffer for 30 minutes, and immunostained overnight at 4 °C with mouse monoclonal antibody to Ndufs4 (1:1000, Abcam) and rabbit polyclonal antibody to beta-Actin (1:5000, Abcam). Membranes were washed 6 times in phosphate-buffered saline (PBS) with 0.1% Tween 20, then immunostained with Goat anti-mouse IgG (H+L) Dylight 680 Conjugated (1:10,000, Thermo Scientific) and goat anti-rabbit IgG (H+L) Dylight 800 Conjugated (1:10,000, Thermo Scientific) secondary antibodies for 1 hour at room temperature. The membrane was washed 6 times in PBS with 0.1% Tween 20, followed by a final wash in PBS.

Membranes were imaged on a Licor Odyssey Blot Imager. The integrated intensity of the Ndufs4 band was normalized to the integrated intensity of the beta-Actin band.

## Ndufs4 gene expression

### RNA isolation

RNA was isolated from the hippocampus using TRIzol (#15596026, Thermo Scientific, Waltham, USA). Briefly, 1 ml of TRIzol was added to each sample and homogenized using a VDI12 tissue homogenizer (VWR, Radnor, USA; 10 seconds per sample, speed setting 6, homogenizer tip was cleaned with MQ and 70% ethanol between samples). After homogenizing, samples were incubated at room temperature for 5 minutes following the addition of 200 µl pure chloroform. The samples were shaken by hand for 15 seconds and were incubated again at room temperature for 5 minutes. After centrifugation at 12.000g for 15 minutes at 4 °C, the aqueous phase was aspirated and put in a new tube. For precipitation of the RNA, 500 µl of pure 2-propanol was added to the aqueous phase. The tubes were shaken for 15 seconds and incubated at room temperature for 15 minutes and centrifuged again. The pellet was washed with 1 ml 75% ethanol and centrifuged at 7.500g for 5 minutes at 4 °C, and the supernatant was discarded. The pellet (RNA) was left to dry for 15 minutes at room temperature and dissolved in 25 µl Ultrapure DNase/RNase-free distilled water (#10977035, Thermo Scientific). The mixture was put in a Thermomixer (Eppendorf, Hamburg, Germany) set to 60 °C (no shaking) for 10 minutes. The RNA concentration was measured in duplicate using a NanoDrop 2000 (Thermo Scientific). All samples had a concentration of around 1000 ng/µl.

### DNase treatment

Following RNA isolation, the samples were treated with RNase-free DNase I (RQ1, Promega, Fitchburg, USA) to eliminate any genomic DNA that may be present. 500 ng of RNA was taken per sample, Ultrapure distilled water was added to 8 µl, and 1 µl of DNase buffer and 1 µl of DNase enzyme were added. This was incubated in a Thermomixer set to 37 °C (no shaking) for 30 minutes. After incubation, 1 µl of Stop Solution was added, and the tube was incubated in a Thermomixer set to 65 °C (no shaking) for 10 minutes. The RNA concentration was measured again in duplicate using a NanoDrop. RNA samples were stored at -80 °C until further use.

### cDNA synthesis

Complementary DNA (cDNA) was synthesized using the iScript kit (#1708891, Bio-Rad, Hercules, USA). 200 ng of DNase-treated RNA was used per sample. Ultrapure distilled water was added to 7.5 µl, and 2 µl of Reaction mix and 0.5 µl of reverse transcriptase enzyme were added. The synthesis was performed in a T100 thermal cycler (Bio-Rad) using the following program: 5 minutes at 25 °C, 20 minutes at 46 °C, 1 minute at 95 °C. The cDNA was diluted 1:10 with Ultrapure distilled water and stored at -20 °C until further use.

### qPCR primer design

Primers were designed using Primer3 software (http://primer3.ut.ee/). The cDNA sequence of the gene of interest was used as a template, and the forward or the reverse primer of each set spans an exon-exon junction. Amplicon length was set between 75 and 125 base pairs. The maximum difference in Tm between forward and reverse primer was 1 °C; maximum poly-x was set to 3, GC content between 40% and 60%. Primers were checked for unwanted possible hairpins and dimers using Beacon Designer Free software (http://www.premierbiosoft.com/qOligo/Oligo.jsp?PID=1). Unwanted possible amplification of genomic DNA sequences was checked by using the *in silico* PCR option from UCSC (https://genome.ucsc.edu/cgi-bin/hgPcr?command=start) set to “genome assembly.” No hits were found for all primers used. Finally, the sequence of the projected amplicon was checked for single nucleotide polymorphisms (SNPs) using the Mouse BLAT Search option from UCSC (https://genome.ucsc.edu/cgi-bin/hgBlat). No known SNPs were found in binding sites of all primers.

### qPCR primer testing

qPCR primers that were designed using the protocol above were ordered from Biolegio (Nijmegen, the Netherlands) or Sigma-Aldrich (St. Louis, USA). 100 µM stocks were diluted into 10 µM working solutions. Amplification efficiency and linearity were assessed by doing qPCR (see below for products used and protocol) on a serial dilution (1:1, 5 steps) of a mixture of cDNA from all samples and plotting the resulting Ct values against the dilution step. A linear trend line was plotted along the graph points, and the equation of this trend line gave the efficiency of the amplification and the R^2^ the linearity. The efficiency of all primer pairs was 90%-105% and linearity >0.99. All primer pairs showed a single peak in the melt curve.

### qPCR

qPCRs were done in 96-well plates (#N8010560, Thermo Scientific) using a 7900HT system (Thermo Scientific). In each well, 5 µl iTaq Universal SYBR Green Supermix (#1725124, Bio-Rad), 3 µl Ultrapure distilled water containing 400 nM of each primer, and 2 µl diluted cDNA was added. Each sample was run in duplicate on the plate. A no-template control (NTC) containing 2 µl Ultrapure distilled water instead of cDNA was also run on the plate in duplicate. The PCR program used was: 30 seconds at 95 °C, 40 cycles of 15 seconds at 95 °C and 1 minute at 60 °C. After that, a melt curve analysis (standard protocol from the machine) was performed. After the run, the threshold was set to 0.2, and the Ct values exported to Microsoft Excel for further analysis. A difference between duplicate values of less than 0.5 Ct was taken as acceptable. For the NTC, no Ct value was seen, or Ct >36 and a Tm value different than the one found for all samples.

The relative expression of *Ndufs4* was determined using two housekeeping genes, *Gapdh* and *B2m*, according to Pfaffl ^10^.

**Table S2**. Forward and reverse primer sequences used for qPCR.

| **Gene** | **RefSeq** | **Forward primer sequence** | **Reverse primer sequence** |
| --- | --- | --- | --- |
| *Ndufs4* | NM_010887.2 | 5’-GTCAGTGTCGCTGAGACAGG-3’ | 5’-AAGCTGTGTGTCCCGAGTCT-3’ |
| *Gapdh* | NM_008084.3 | 5’-GTCGGTGTGAACGGATTTGG-3’ | 5’-ACAATCTCCACTTTGCCACTG-3’ |
| *B2m* | NM_009735 | 5’-GATGTCAGATATGTCCTTCAGCA-3’ | 5’-TCACATGTCTCGATCCCAGT-3’ |

## Blood plasma proteins

To determine the concentration of different proteins in the blood plasma, several commercially available ELISAs were used. The plasma blood levels of insulin (Crystal Chem Inc., protocol v7) and corticosterone (CORT; Abcam, protocol v4) were analyzed. All samples were assayed in duplicate according to the manufacturer's protocol.

## Tissue processing for immunocytochemistry

From half of the animals, the right hemisphere was immersion fixated in 4% formaldehyde at 4 °C for approximately 20 hours. After 20 hours, the brains were stored in PBS with 0.01% NaN_3_ at 4 °C until sectioning. The brains were coronally sectioned using a Leica VS1000 vibratome (Leica Biosystems, Wetzlar, Germany). Five series of 30 µm thick sections were collected and stored in PBS with 0.01% NaN_3_ and stored at 4 °C until further use.

## Immunohistochemical stainings

Ionized calcium-binding adapter molecule 1 (IBA-1) and doublecortin (DCX) stainings were performed in the hippocampus to determine the amount of activated microglia and neurogenesis, respectively. For both stainings, a standard free-floating immunohistochemistry protocol was used. All incubations were performed at room temperature on a shaker table. In short, approximately ten sections of the dorsal hippocampus (Bregma level -0.58 till -2.30) were selected per animal. Sections were washed three times 10 minutes with 0.1M PBS pH 7.3, followed by a 30-minute incubation in PBS containing 0.3% H_2_O_2_ to block endogenous peroxidase activity. After washing, the sections were pre-incubated in PBS-BT (PBS with 0.1% Bovine serum albumin and 0.3% Triton X-100) to block non-specific binding sites and increase the permeability of the tissue. Following this pre-incubation, sections were incubated overnight in either primary polyclonal goat anti-IBA-1 [1:4000] (Abcam) or polyclonal goat anti-DCX (C18) [1:4000] (Santa Cruz) in PBS-BT. After incubation, sections were washed and incubated in polyclonal biotinylated donkey anti-goat secondary antibody [1:1500] (Jackson ImmunoResearch) in PBS-BT for 90 minutes. After washing, the sections were incubated for 90 minutes in Vector ABC-Elite (Vector Laboratories, Burlingame, CA, USA) [1:800] in PBS-BT, followed by another wash step. The reaction was visualized by pre-incubation for 10 minutes in 3-3’diaminobenzidine tetrahydrochloride (Sigma-Aldrich) with 0.3% ammonium nickel sulfate as an intensifier (DAB-Ni, pH 7.6) without H2O2 followed by incubation in DAB-Ni with 0.006% H2O2 for 10 min. After precisely 10 minutes, the reaction was stopped by washing in PBS; the sections were mounted on gelatin-coated glass slides (0.5% gelatin and 0.05% chrome-alum sulfate) and dried overnight at 37 °C. The slides containing the sections were dehydrated via gradual ethanol steps of each 2 minutes (50%, 70%, 3x 96%, 3x 100%) followed by three times Xylene for 2 minutes. As the last step, the sections were coverslipped in Entallan (Merck KGaA, Darmstadt, Germany).

### Image acquisition

For the IBA-1 and DCX stainings, three sections per animal were selected for quantification around Bregma -1.20, -1.45, and -1.80 based on the Franklin and Paxinos mouse brain atlas third edition ^11^. Digital images of the IBA-1 staining were made using a Zeiss Axio Imager A2 equipped with a monochrome camera (Axiocam ICm1) at 5x magnification. The DCX positive neurons were counted manually with a Zeiss Axioskop microscope equipped with hardware and software from Microbrightfield (Williston, VT, USA). On each Bregma, two areas were counted, the subgranular zone of the dentate gyrus and the subventricular zone (SVZ) to investigate the migration of new neurons. Measurements were averaged to obtain a single value per animal.

### Image analysis

Digital images of the IBA-1 staining were imported in ImageJ software, converted to 8-bit, and a threshold was set for each image individually, so most stained microglia could be counted automatically by the program in the whole hippocampus. DCX positive cells were counted manually using the Microbrightfield hardware and software, as described above.

## *Ex vivo* magnetic resonance diffusion tensor imaging

### Acquisition

The brains of five WT and five *Ndufs4*^GT/GT^ 10-week-old mice were used to study structural differences using high-resolution *ex vivo* magnetic resonance diffusion tensor imaging (DTI). Animals were killed by cervical dislocation, and the brains were removed and immersion fixated in 4% formaldehyde for more than 4 weeks at 4 °C. Before magnetic resonance scanning, the brains were rehydrated in PBS for 2 weeks to reduce fixation induced T2 shortening ^12^. 24 hours before the scan, the brains were transferred in a 5 ml syringe containing 3M Fluorinert FC-3283. The brain was immobilized in the syringe with gauze, and all the air bubbles were removed using a slight vacuum and stored at 4 °C until scanned. Magnetic resonance (MR) measurements were performed on an 11.7 T BioSpec Avance III small-animal MR system (Bruker BioSpin, Ettlingen, Germany) operating on ParaVision 6.0.1 software platform (Bruker, Karlsruhe, Germany). A circular polarized resonator was used for signal transmission and an actively-decoupled mouse brain quadrature surface coil for signal reception (Bruker BioSpin). Gradient echo images in the axial, sagittal, and coronal orientation were acquired to visualize anatomy and morphology with a slightly modified protocol according to Zerbi*, et al.* ^13^. In short, imaging parameters were: TE=5 ms, TR=3320.283ms, flip angle=30^o^, field of view=16x16 mm, matrix size = 64x64, slice thickness = 0.25 mm, 55 slices creating isotropic voxels with a total scan time of 3.32 minutes.

Diffusion-weighted images were acquired using a modified protocol according to Kleinnijenhuis*, et al.* ^14^. In short, 55 axial slices covering the whole brain, excluding the olfactory bulb, were acquired with a spin-echo planar imaging protocol using segmented echo-planar imaging (EPI) readout (one segment). Diffusion gradients were applied with a B-value of 1144.4 sec/mm^2^. These diffusion gradients were applied to 768 non-collinear directions. B0 shift compensation, navigator echoes, and automatic ghost correction algorithm were implemented to limit the occurrence of ghosts and artifacts. Other imaging parameters were: TE=31 ms, TR=10.000 ms, field of view=16x16mm, matrix size=64x64, slice thickness=0.25mm, 55 slices creating isotropic voxels with a total scan time of 9:15 hours per mouse brain.

### Analysis

From each mouse the fractional anisotropy (FA), mean water diffusivity (MD), radial diffusivity (RD), and axial diffusivity (AD) were derived from the tensor estimation following a protocol as described elsewhere ^13^. MD is considered an inverse measure of membrane density and is sensitive to changes in gray matter (GM), while FA is an estimate of myelination and fiber density in white matter (WM) ^15-17^. FA, MD, RD, and AD values were measured in several WM and GM areas that were manually selected based on the Franklin and Paxinos mouse brain atlas ^11^.

## Brain-targeted metabolomics

From the left, snap frozen, brain hemisphere, prefrontal cortices were dissected. These were used to measure several different metabolites from the tricarboxylic acid cycle (TCA), acyl-carnitines (AC), as well as amino acids (AA).

The concentrations of several TCA cycle analytes were measured by gas chromatograph mass spectrometry (GC/MS), as previously described with a few modifications ^18,19^. Briefly, 10 mg of pulverized tissue was homogenized in PBS before adding 15 µl of internal solution containing U-^13^C labeled analytes. The proteins were removed by adding 460 µl of chilled methanol and acetonitrile solution to the sample mixture. After drying the supernatant in a speed vac, the sample was derivatized with ethoxime and then with MtBSTFA + 1% tBDMCS (N-Methyl-N-(t-Butyldimethylsilyl)-Trifluoroacetamide + 1% t-Butyldimethylchlorosilane) before it was analyzed on an Agilent 5977B GC/MS (gas chromatography/mass spectrometry) under electron impact and single ion monitoring conditions. Concentrations of lactic acid (m/z 261.2), fumaric acid (m/z 287.1), succinic acid (m/z 289.1), oxaloacetic acid (m/z 346.2), ketoglutaric acid (m/z 360.2), malic acid (m/z 419.3), aspartic acid (m/z 418.2), 2-hydroxyglutaratic acid (m/z 433.2), cis-aconitic acid (m/z 459.3), citric acid (m/z 591.4), isocitric acid (m/z 591.4), and glutamic acid (m/z 432.4) were measured against a 7-point calibration curves that underwent the same derivatization. Oxaloacetate and a few samples for 2-Hydroxyglutarate were not included in the analysis because the concentrations were below the limit of quantitation (LOQ).

The concentrations of acyl-carnitines (AC) were measured with a UPLC-MS method, as previously described ^20^. In short, 5 mg of pulverized tissue was homogenized in 50 µl of PBS before 25 µl of deuterated labeled internal standards was added. Proteins were removed by adding a solution of methanol/dichloromethane (v/v, 600 µl) to the sample mixture. The sample was centrifuged at 18.000g for 15 mins at 4 °C, supernatant transferred to a 1 dram vial, and dried under N_2_ stream. Samples were reconstituted and analyzed on a Waters Acquity UPLC system (Milford, MA) coupled with a Thermo Quantiva tandem mass spectrometer (West Palm Beach, FL) in positive (H)ESI mode. Concentrations of carnitine (162.1>85.0 m/z), acetylcarnitine (204.1>85.0 m/z), propionylcarnitine (218.1>85.0 m/z), butyrylcarnitine (232.1>85.0 m/z), isovalerylcarnitine (246.1>85.0 m/z), octanoylcarnitine (288.2>85.0 m/z), lauroylcarnitine (344.3>85.0m/z), myristoylcarnitine 372.3> 85.0 m/z), palmitoylcarnitine (400.4>85.0 m/z), oleoylcarnitine (426.4>85.0m/z), and stearoylcarnitine (438.4>85.0m/z) were measured against a 11-point calibration curve that underwent the same preparation.

Amino acids (AA) and their metabolites were measured by LCMS, as previously described ^21^. Briefly, 5 mg of pulverized tissue was homogenized in PBS prior to adding internal solution containing U-13C labeled analytes. The sample mixture was sonicated and deproteinized with cold methanol followed by centrifugation at 18.000g for 15 minutes. The supernatant was dried down and then derivatized with 6-aminoquinolyl-N-hydroxysuccinimidyl carbamate, according to Waters’ MassTrak kit. An 11-point calibration standard curve underwent similar derivatization procedure after the addition of internal standards. Both derivatized standards and samples were analyzed on a Thermo TSQ Quantum Ultra mass spectrometer (West Palm Beach, FL) coupled with a Waters Acquity UPLC system (Milford, MA). Data acquisition was done using select ion monitor (SRM). Concentrations of 42 analytes were calculated against their perspective calibration curve. Some metabolites were not included in the analysis because concentrations were either below the limit of quantitation (LOQ, 1-methylhistidine, 3-methylhistidine, and sarcosine) or over the curve (taurine).

## Statistics

For statistical analysis, IBM SPSS 24 software (IBM Corporation, New York, NY, USA) was used. Five animals were removed from all analyses. Two stressed *Ndufs4*^GT/GT^ mice showed stereotypical behavior, two WT animals had a severely impaired mitochondrial CI activity whilst one *Ndufs4*^GT/GT^ animal had very high mitochondrial CI activity. To determine differences between genotype (WT or *Ndufs4*^GT/GT^) and condition (control or stressed), two-way ANOVAs were used after testing for normality using Shapiro-Wilk tests as well as the Levene’s test for equality of error variances. When a statistically significant interaction was found between genotype and condition, the ANOVA was rerun with simple effects using a Bonferroni correction. When appropriate, repeated measures ANOVAs were performed. When only the genotype was assessed, Student’s t-tests were used to determine statistical significance. Detailed outcomes of the statistical tests and the sample sizes are summarized in **Supplementary** **Table 1**. Statistical significance was set at P<0.05, whereas a statistical trend was set at P<0.07. All data are presented as mean ± SEM.

# Supplementary References

1 Pugh P.L., Ahmed S.F., Smith M.I., Upton N. & Hunter A.J. A behavioural characterisation of the FVB/N mouse strain. *Behav. Brain Res.* **155**, 283-289, doi:10.1016/j.bbr.2004.04.021 (2004).

2 Giles J.M., Whitaker J.W., Moy S.S. & Fletcher C.A. Effect of Environmental Enrichment on Aggression in BALB/cJ and BALB/cByJ Mice Monitored by Using an Automated System. *Journal of the American Association for Laboratory Animal Science* **57**, 236-243, doi:10.30802/Aalas-Jaalas-17-000122 (2018).

3 Rodenburg R.J. Biochemical diagnosis of mitochondrial disorders. *J. Inherit. Metab. Dis.* **34**, 283-292, doi:10.1007/s10545-010-9081-y (2011).

4 Cooperstein S.J. & Lazarow A. A microspectrophotometric method for the determination of cytochrome oxidase. *J. Biol. Chem.* **189**, 665-670 (1951).

5 Janssen A.J. *et al.* Spectrophotometric assay for complex I of the respiratory chain in tissue samples and cultured fibroblasts. *Clin. Chem.* **53**, 729-734, doi:10.1373/clinchem.2006.078873 (2007).

6 Mourmans J. *et al.* Clinical heterogeneity in respiratory chain complex III deficiency in childhood. *J. Neurol. Sci.* **149**, 111-117 (1997).

7 Srere P. Citrate synthase:[EC 4.1. 3.7. Citrate oxaloacetate-lyase (CoA-acetylating)]. *Methods Enzymol.* **13**, 3-11 (1969).

8 Fischer J.C. *et al.* Differential investigation of the capacity of succinate oxidation in human skeletal muscle. *Clin. Chim. Acta* **153**, 23-36 (1985).

9 Janssen A.J. *et al.* Measurement of the energy-generating capacity of human muscle mitochondria: diagnostic procedure and application to human pathology. *Clin. Chem.* **52**, 860-871, doi:10.1373/clinchem.2005.062414 (2006).

10 Pfaffl M.W. A new mathematical model for relative quantification in real-time RT–PCR. *Nucleic Acids Res.* **29**, e45-e45 (2001).

11 Paxinos G. & Franklin K.B. *The Mouse Brain in Stereotaxic Coordinates Third Edition*. (Elsevier Academic Press, 2007).

12 Shepherd T.M., Thelwall P.E., Stanisz G.J. & Blackband S.J. Aldehyde fixative solutions alter the water relaxation and diffusion properties of nervous tissue. *Magn. Reson. Med.* **62**, 26-34, doi:10.1002/mrm.21977 (2009).

13 Zerbi V. *et al.* Gray and white matter degeneration revealed by diffusion in an Alzheimer mouse model. *Neurobiol. Aging* **34**, 1440-1450, doi:10.1016/j.neurobiolaging.2012.11.017 (2013).

14 Kleinnijenhuis M. *et al.* Layer-specific diffusion weighted imaging in human primary visual cortex in vitro. *Cortex* **49**, 2569-2582, doi:10.1016/j.cortex.2012.11.015 (2013).

15 Le Bihan D. *et al.* Diffusion tensor imaging: concepts and applications. *J. Magn. Reson. Imaging* **13**, 534-546 (2001).

16 Alexander A.L. *et al.* Characterization of cerebral white matter properties using quantitative magnetic resonance imaging stains. *Brain Connect.* **1**, 423-446 (2011).

17 Feldman H.M., Yeatman J.D., Lee E.S., Barde L.H. & Gaman-Bean S. Diffusion tensor imaging: a review for pediatric researchers and clinicians. *J. Dev. Behav. Pediatr.* **31**, 346-356, doi:10.1097/DBP.0b013e3181dcaa8b (2010).

18 Dutta T. *et al.* Impact of Long-Term Poor and Good Glycemic Control on Metabolomics Alterations in Type 1 Diabetic People. *J. Clin. Endocrinol. Metab.* **101**, 1023-1033, doi:10.1210/jc.2015-2640 (2016).

19 Koek M.M., Muilwijk B., van der Werf M.J. & Hankemeier T. Microbial metabolomics with gas chromatography/mass spectrometry. *Anal. Chem.* **78**, 1272-1281 (2006).

20 Chace D.H. *et al.* Electrospray tandem mass spectrometry for analysis of acylcarnitines in dried postmortem blood specimens collected at autopsy from infants with unexplained cause of death. *Clin. Chem.* **47**, 1166-1182 (2001).

21 Lanza I.R. *et al.* Quantitative metabolomics by 1H-NMR and LC-MS/MS confirms altered metabolic pathways in diabetes. *PLoS One* **5**, e10538 (2010).

# Supplementary Figure Legends

## **Fig. S1**. Schematic overview of the chronic unpredictable stress (CUS) paradigm with the list of stressors and behavioral tests used. (**A**) A schematic representation of the whole experiment from start to finish is shown. Minimally ten days before the start of the CUS paradigm, at 9 weeks of age, the mice arrived at the animal facility to acclimatize. At day zero, the CUS started until day 21. (**B**) Animals in the stress group were subjected to the CUS paradigm consisting of different once-daily physical and psychological stressors. (**C**) During the study, both control and stressed animals were subjected to different behavioral tests. Approximately half of the animals were subjected to the forced swim test, Rotarod, open field, and splash test while the other half of the animals were subjected to the tail suspension test, grip test, elevated plus maze, and sucrose preference test. On day 20, only stressed mice received either the FST or TST. On day 22, all mice were sacrificed, and biological material was collected.

## **Fig. S2.** *Ndufs4*^GT/GT^ mice only showed a decreased mitochondrial complex I (CI) activity, while stress does not influence any of the mitochondrial oxidative phosphorylation (OXPHOS) enzyme activities. (**A**) All mitochondrial complex activities, maximal ATP production rate, as well as citrate synthase activity, a marker for mitochondrial abundance, were measured in WT, heterozygous (HET), and *Ndufs4*^GT/GT^ mice. Data from each measurement was normalized to WT. (**B**) The same measurements were performed in WT and *Ndufs4*^GT/GT^ mice that were subjected to chronic stress or served as controls. Each measurement was normalized to WT control. Graphs show average ± SEM. * p<0.05, ** p<0.01, and *** p<0.001. Abbreviations: CI, mitochondrial complex I; CII, complex II; CIII, complex III, COX, cytochrome c oxidase (CIV); SCC, succinate: cytochrome c oxidoreductase (measuring CII -> CoQ10 -> CIII); CS, citrate synthase.

## **Fig. S3**. There are no structural differences in different brain regions between 10-week-old WT and Ndufs4^GT/GT^ mice, as assessed with high-resolution ex vivo diffusion tensor imaging (DTI). (**A**) Axial diffusivity (AD) was measured in different brain regions. (**B**) In the same brain regions radial diffusivity (RD) was measured. Graphs show average ± SEM. Abbreviations: AC, anterior commissure; CC, corpus callosum; Cereb, cerebellum; CP, cerebral peduncle; CPu, caudate putamen; CTX, cortex; dHip, dorsal part of the hippocampus; IC, internal capsule; Mid, midbrain; Sub, subthalamus; vHip, ventral part of the hippocampus.

## **Fig. S4**. A lower mitochondrial function in Ndufs4^GT/GT^ mice resulted in slightly lower body weight, while stress impaired the body weight gain. (**A**) From weening onward, the body weight was measured in both WT and Ndufs4GT/GT mice every week. The average difference in body weight is also depicted per week (yellow line). (**B**) All animals were weighed every five days starting ten days before the chronic stress induction to assess body weight change throughout the experiment. Day -10 was set to 100%. (C) The average weight change during chronic stress was calculated from day 0 (start of the stress) to day 20 (end of the stress). Graphs show average ± SEM, with each black dot representing the result of an individual animal. * p<0.05, ** p<0.01, and *** p<0.001.

## **Fig. S5.** Ndufs4 deficiency or chronic stress did not affect insulin concentrations or the insulin/glucose ratio. (**A**) The plasma insulin concentration was measured with an ELISA. (**B**) The insulin/glucose ratio was also calculated to investigate a potential diabetic phenotype of the mice. Graphs show average ± SEM, with each black dot representing the result of an individual animal.

## **Fig. S6.** *Ndufs4*^GT/GT^ mice are slightly more active during the night at baseline. (**A**) Because the animals are housed individually and housed in DVC cages, the home cage activity of each animal was measured continuously before initiation of the chronic unpredictable stress (CUS) paradigm as well as (**B**) after the CUS. Graphs show average ± SEM, with each black dot representing the result of an individual animal. * p<0.05

## **Fig. S7**. No effect of genotype or stress was found on the behavior in the elevated plus maze (EPM). (**A**) Representative locomotion tracks from the EPM. Vertical arms are the open arms, and horizontal arms are the closed arms as indicated. Traces of all animals during EPM are shown in **Fig. S11**. (**B**) The total distance moved in meters was measured in the EPM. (**C**) The ratio between open arm time and total arm time, (**D**) as well as the ratio between open arm entries and total arm entries. The center was excluded from analyses of the EPM. Graphs show average ± SEM, with each black dot representing the result of an individual animal.

## **Fig. S8**. All open field and elevated plus maze (EPM) activity traces. (**A**) The activity traces of all animals during the open field test are shown. Two animals in the WT control group were excluded because of their low complex I (CI) activity and two animals in the *Ndufs4*^GT/GT^ stress group had to be excluded due to stereotypical behavior as also described in the “Statistics” section. (**B**) The activity traces of all animals during the EPM are shown. During the EPM, one WT control and one *Ndufs4*^GT/GT^ stress animal fell off the open arm and had to be excluded. Another *Ndufs4*^GT/GT^ stress animal was excluded due to a high CI activity. The vertical arms are open, and the horizontal arms are closed as indicated.

## **Fig. S9.** All results from the measured AA metabolites ordered alphabetically. Some metabolites are not included because concentrations were either below the limit of quantitation (1-methylhistidine, 3-methylhistidine, and sarcosine), or over the curve (taurine). Data were normalized to total AA concentration and show average ± SEM, with each black dot representing the result of an individual animal. * p<0.05, ** p<0.01, and *** p<0.001

## **Fig. S10.** All results of the acyl-carnitine (AC) metabolites. (**A**) Raw data of the several analyzed acyl-carnitines relative to WT control. (**B**) Calculated ratios of AC metabolites to acylated carnitine relative to WT control. (**C**) Calculated ratios of AC metabolites to free carnitine relative to WT control. (**D**) Calculated ratios of AC metabolites to total carnitine relative to WT control. Graphs show average ± SEM. * p<0.05 and # p<0.07.

## **Fig. S11.** All raw data graphs from the measured TCA metabolites. Oxaloacetate is not included because the concentrations were below the limit of quantitation (LOQ). Data were normalized to total TCA concentration and show average ± SEM, with each black dot representing the result of an individual animal. * p<0.05 and # p<0.07.

# Supplementary Table Legend

## **Supplementary Table 1.** Summary of all statistical analysis used in the study.
