# Supplementary figures and images for: Impaired mitochondrial complex I function as a candidate driver in the biological stress response and a concomitant stress-induced brain metabolic reprogramming in male mice
Source: Transl Psychiatry. 2020 Jun 1;10:176. doi: 10.1038/s41398-020-0858-y (PMC7266820; doi:10.1038/s41398-020-0858-y)

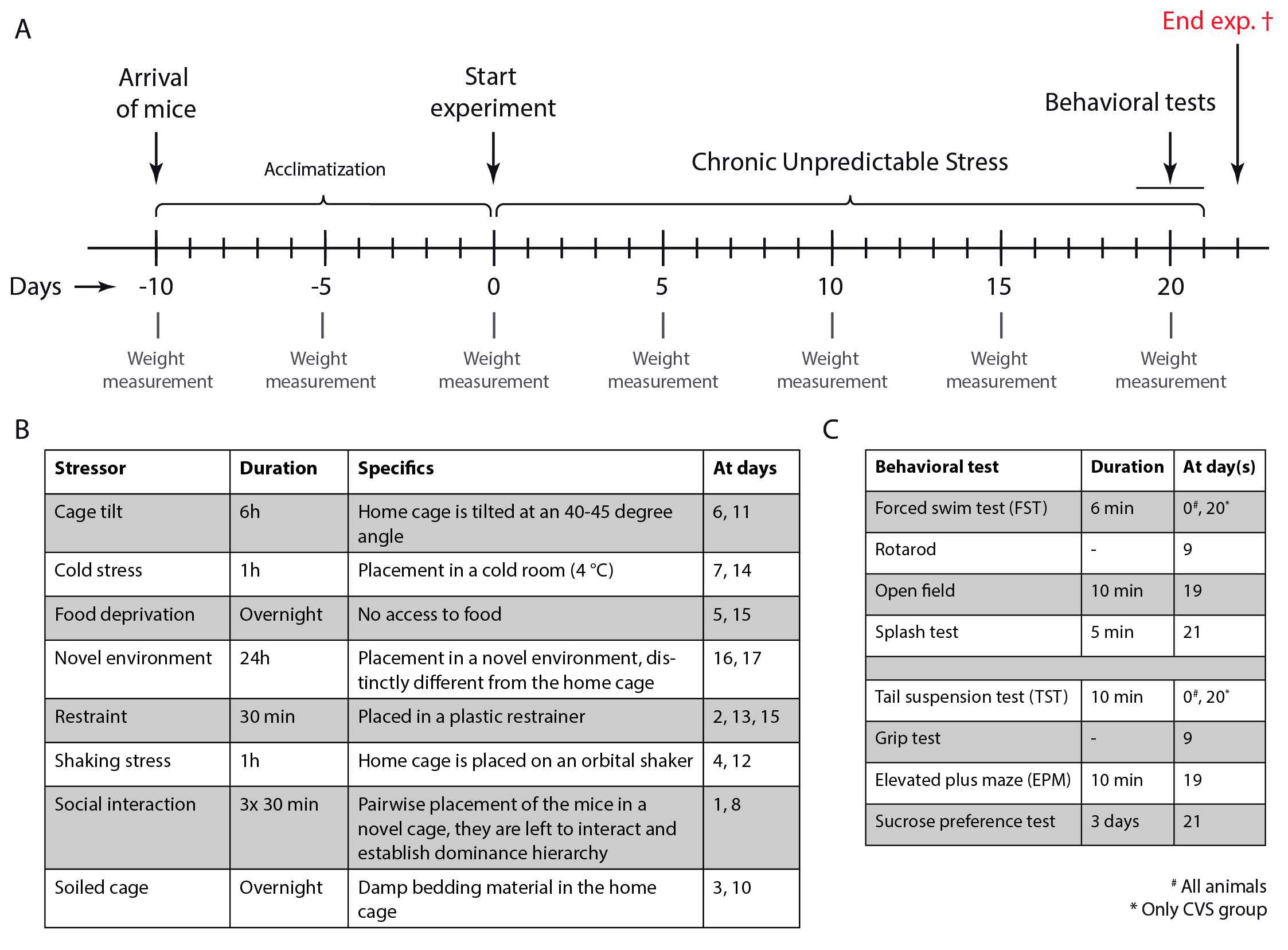

Supplement: Supplementary file 2 — Figure S1 [file 41398_2020_858_MOESM2_ESM.tif]

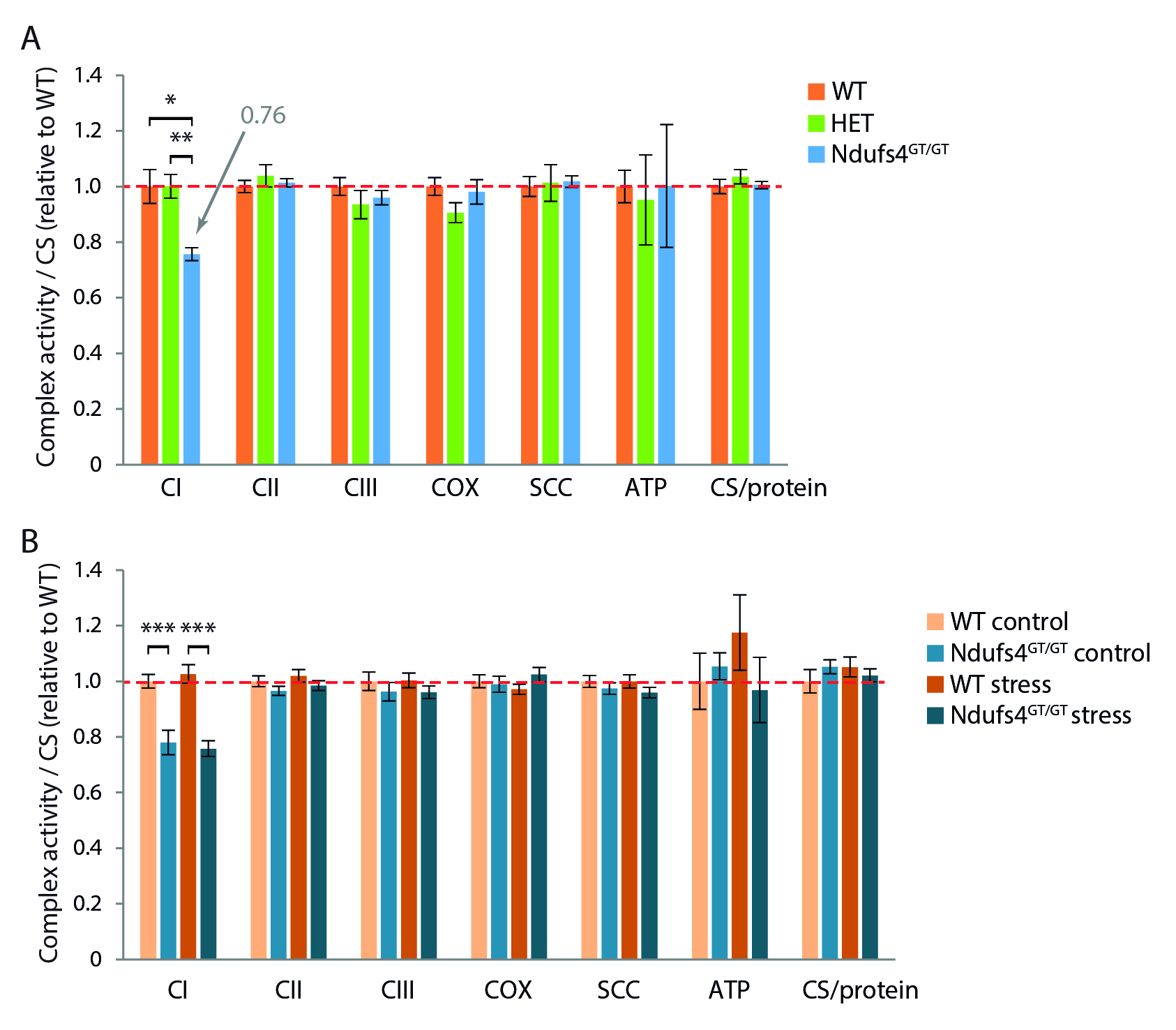

Supplement: Supplementary file 3 — Figure S2 [file 41398_2020_858_MOESM3_ESM.tif]

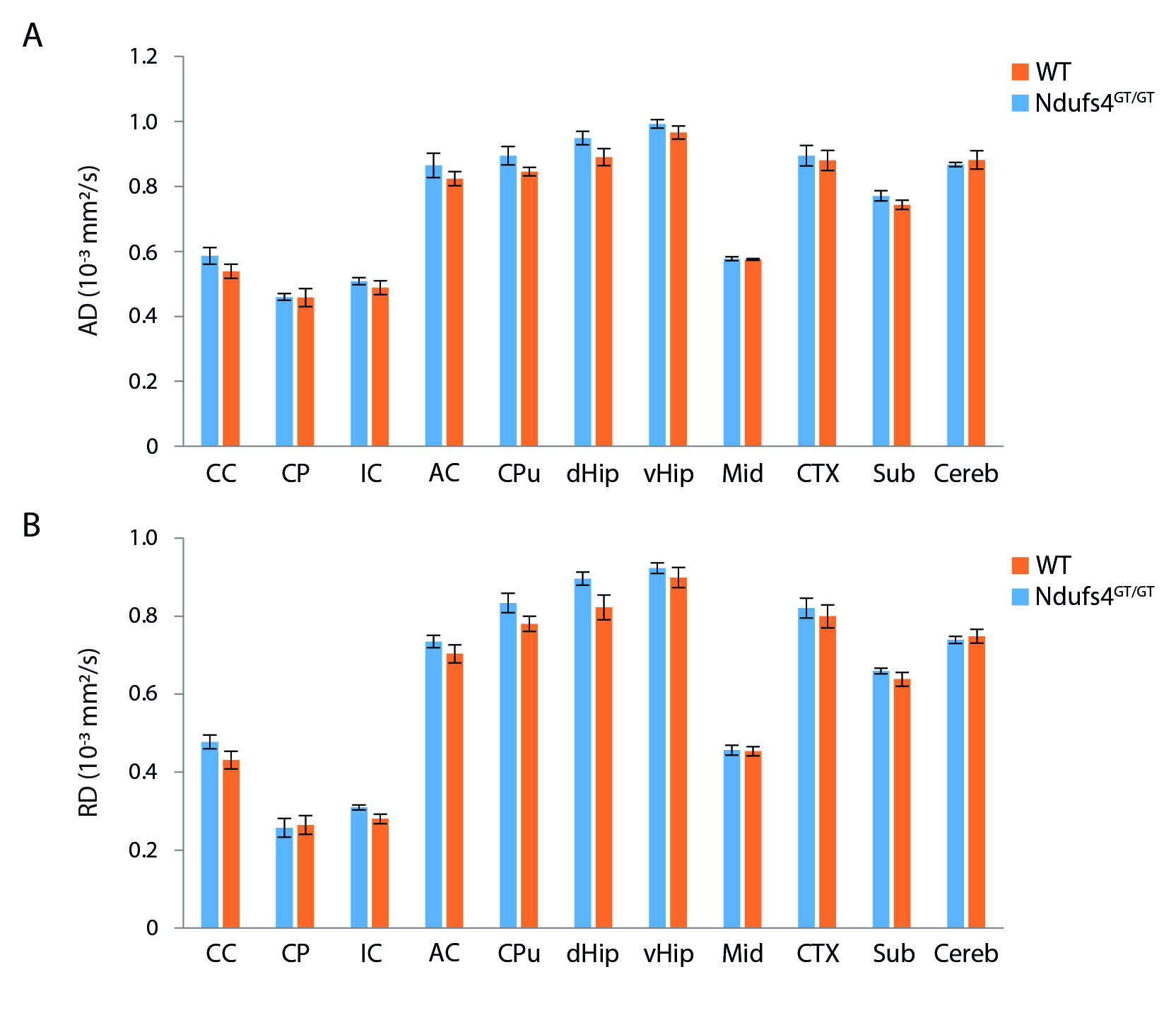

Supplement: Supplementary file 4 — Figure S3 [file 41398_2020_858_MOESM4_ESM.tif]

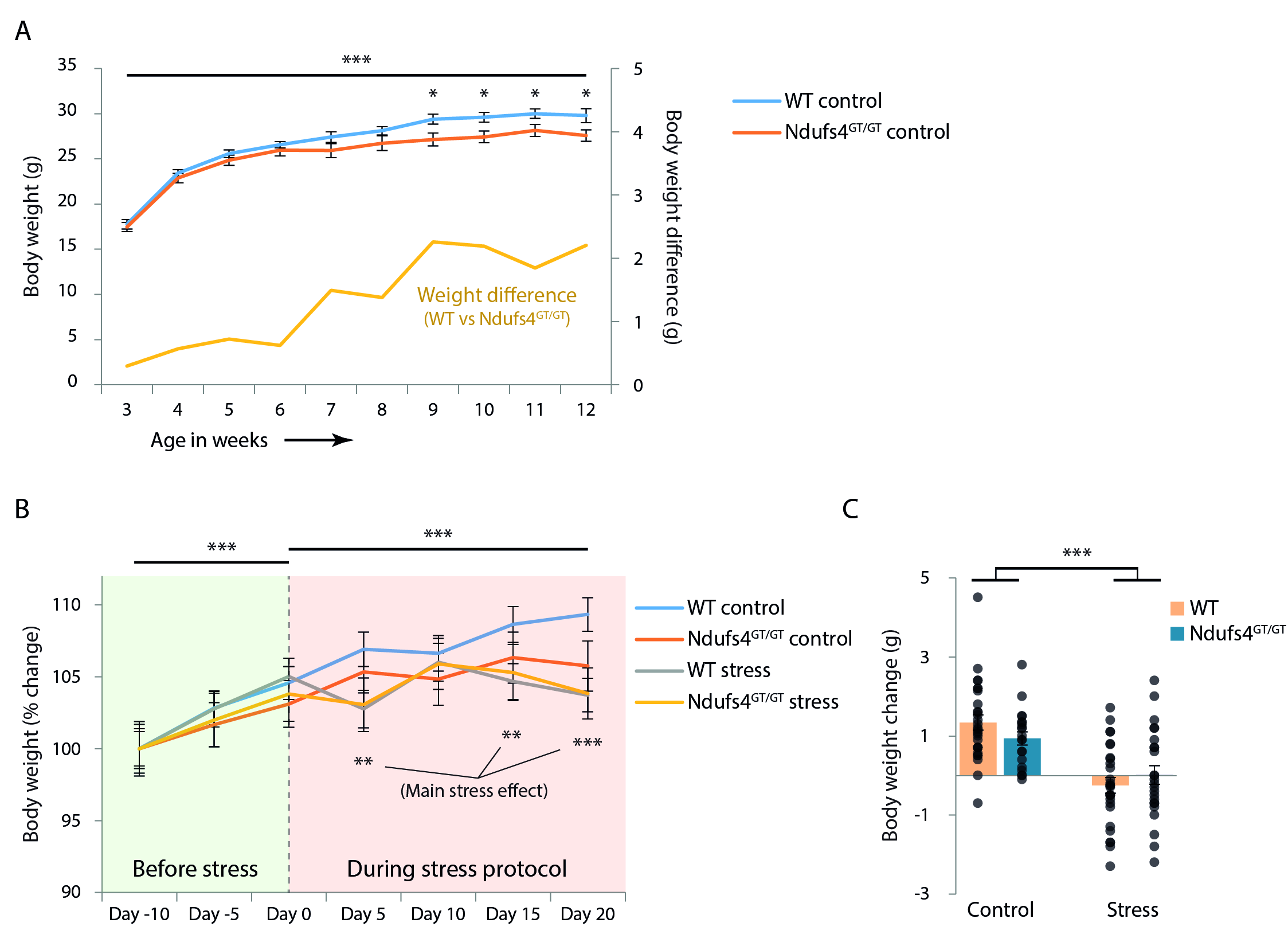

Supplement: Supplementary file 5 — Figure S4 [file 41398_2020_858_MOESM5_ESM.tif]

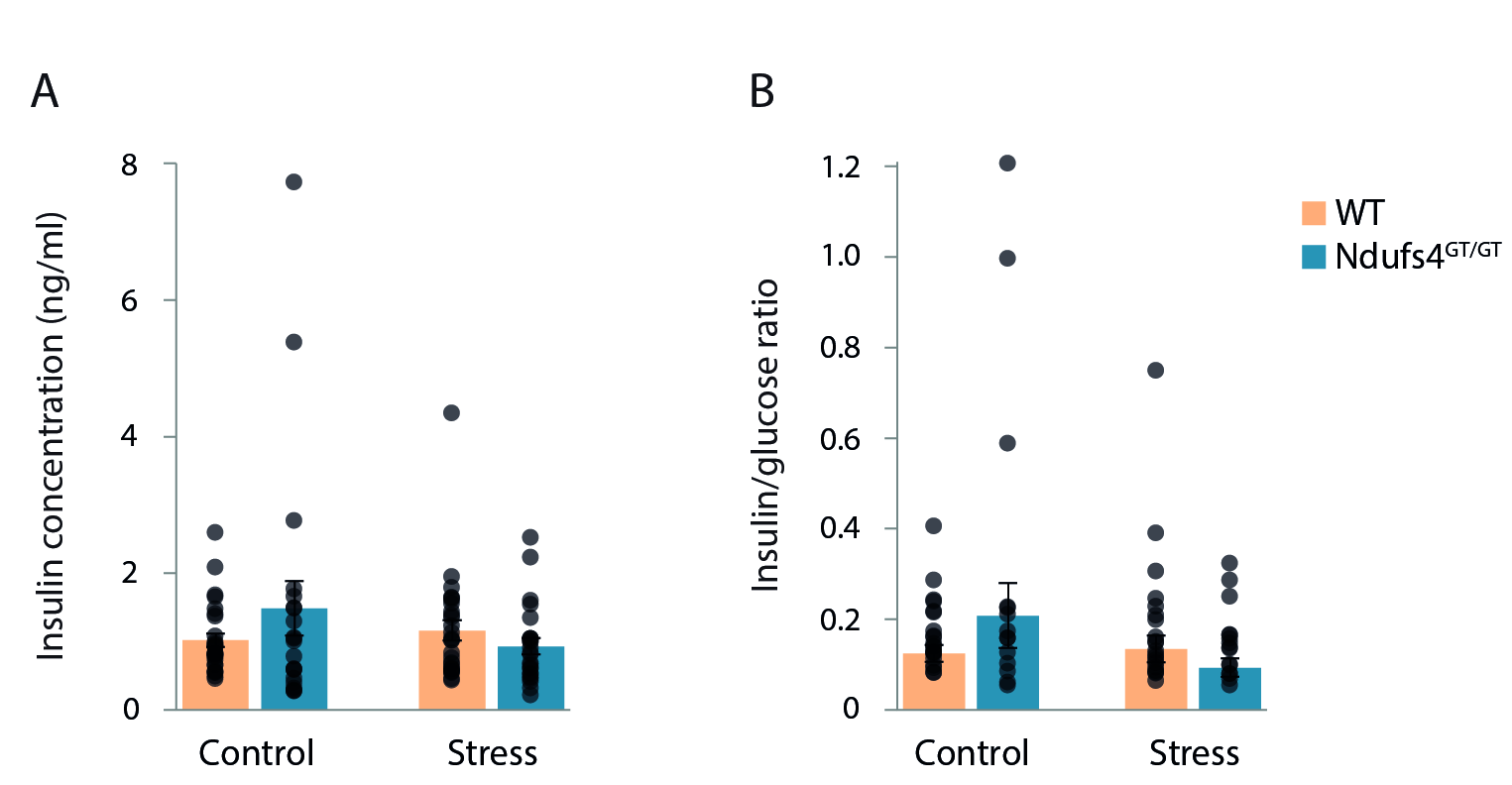

Supplement: Supplementary file 6 — Figure S5 [file 41398_2020_858_MOESM6_ESM.tif]

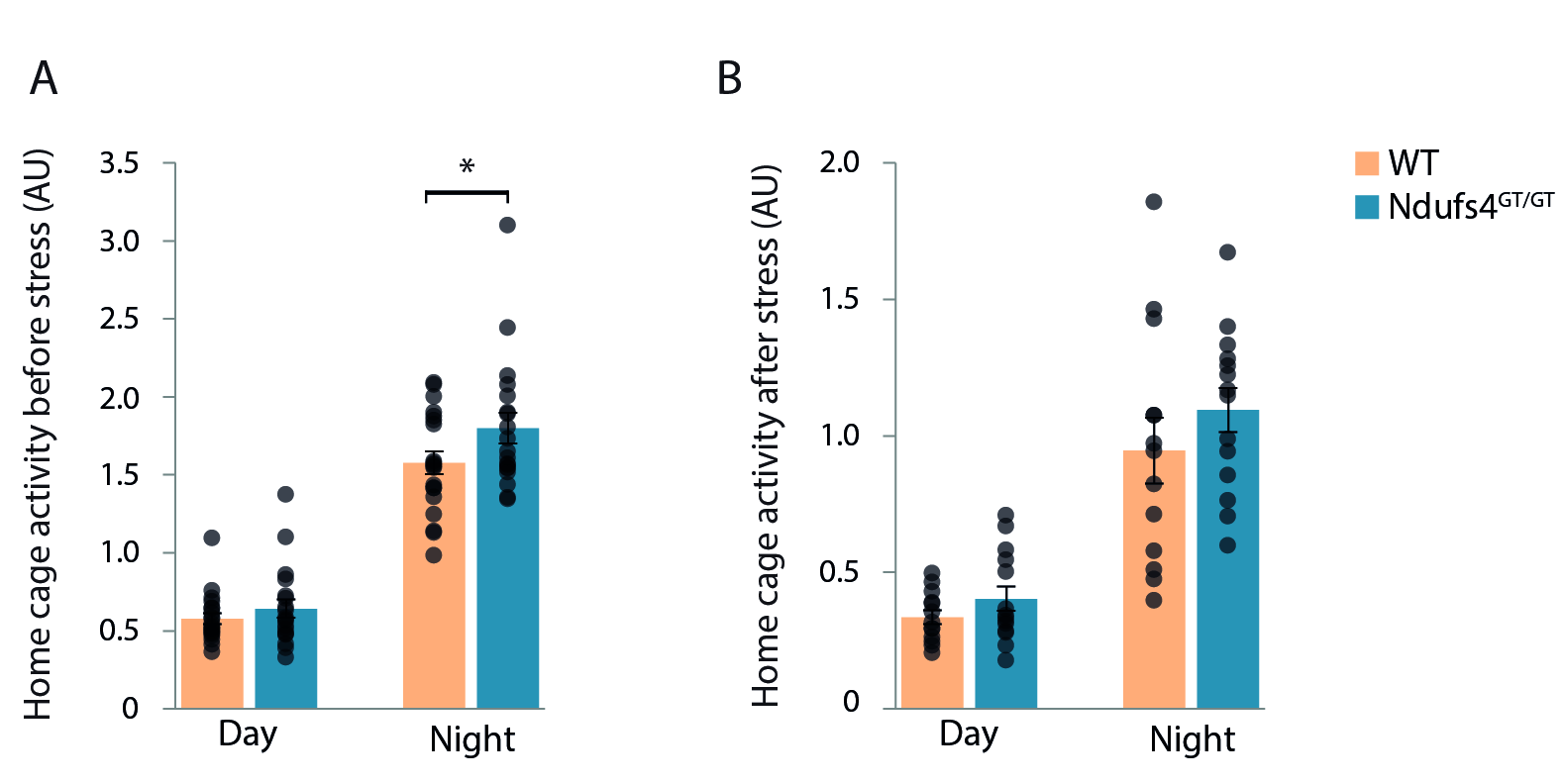

Supplement: Supplementary file 7 — Figure S6 [file 41398_2020_858_MOESM7_ESM.tif]

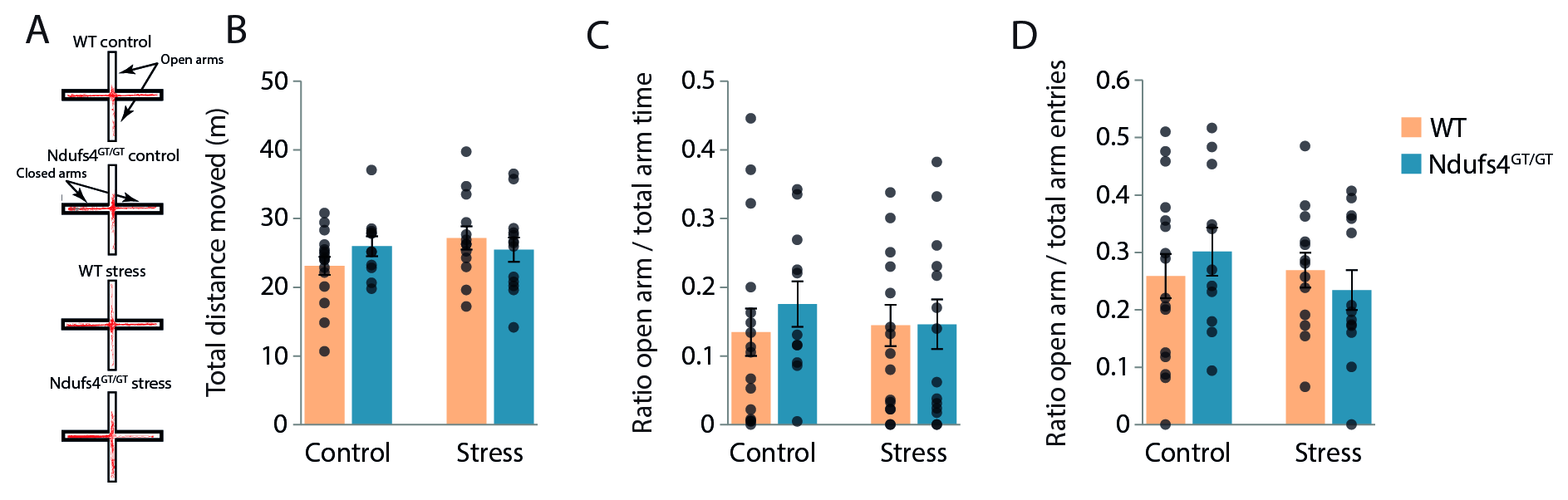

Supplement: Supplementary file 8 — Figure S7 [file 41398_2020_858_MOESM8_ESM.tif]

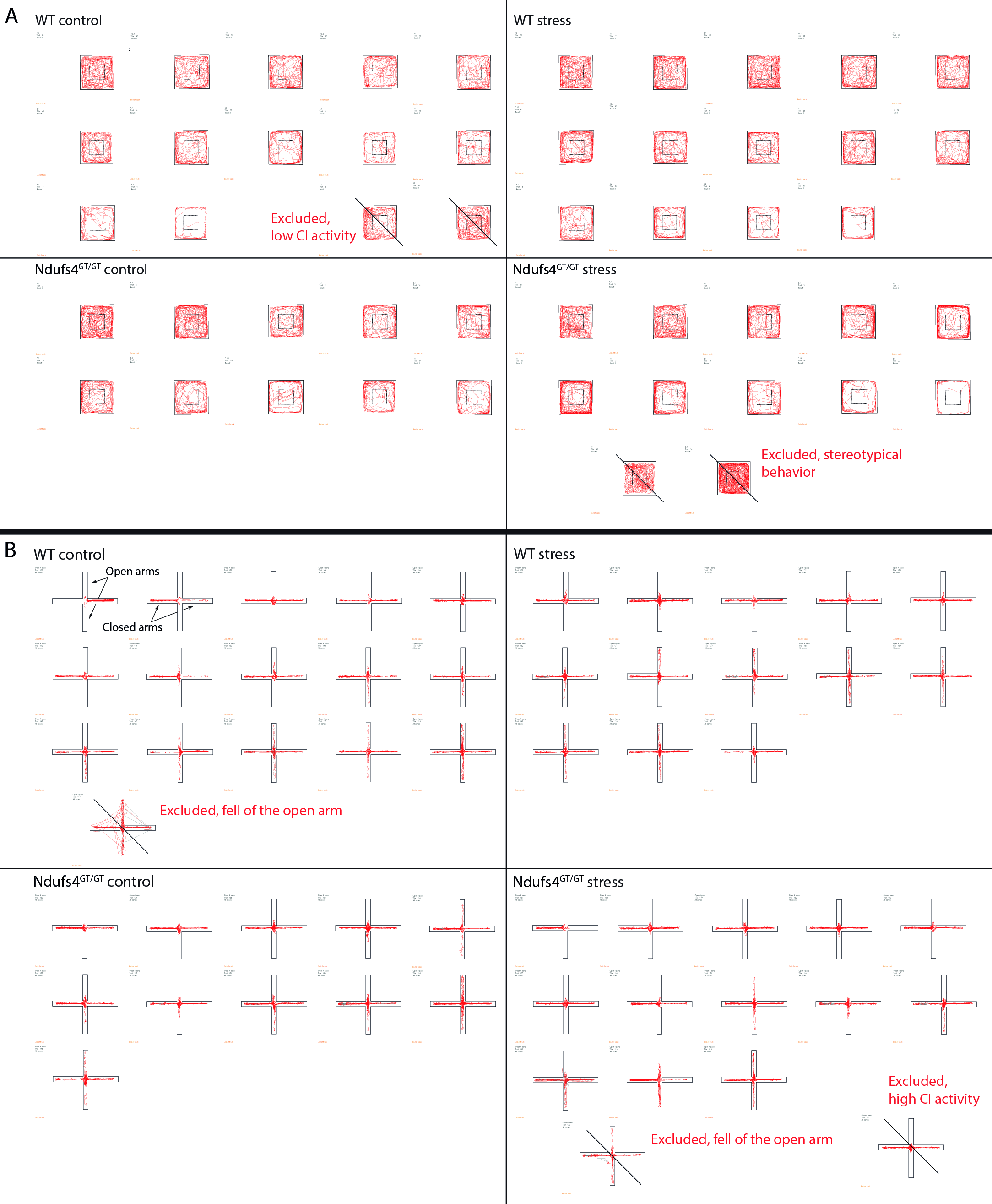

Supplement: Supplementary file 9 — Figure S8 [file 41398_2020_858_MOESM9_ESM.tif]

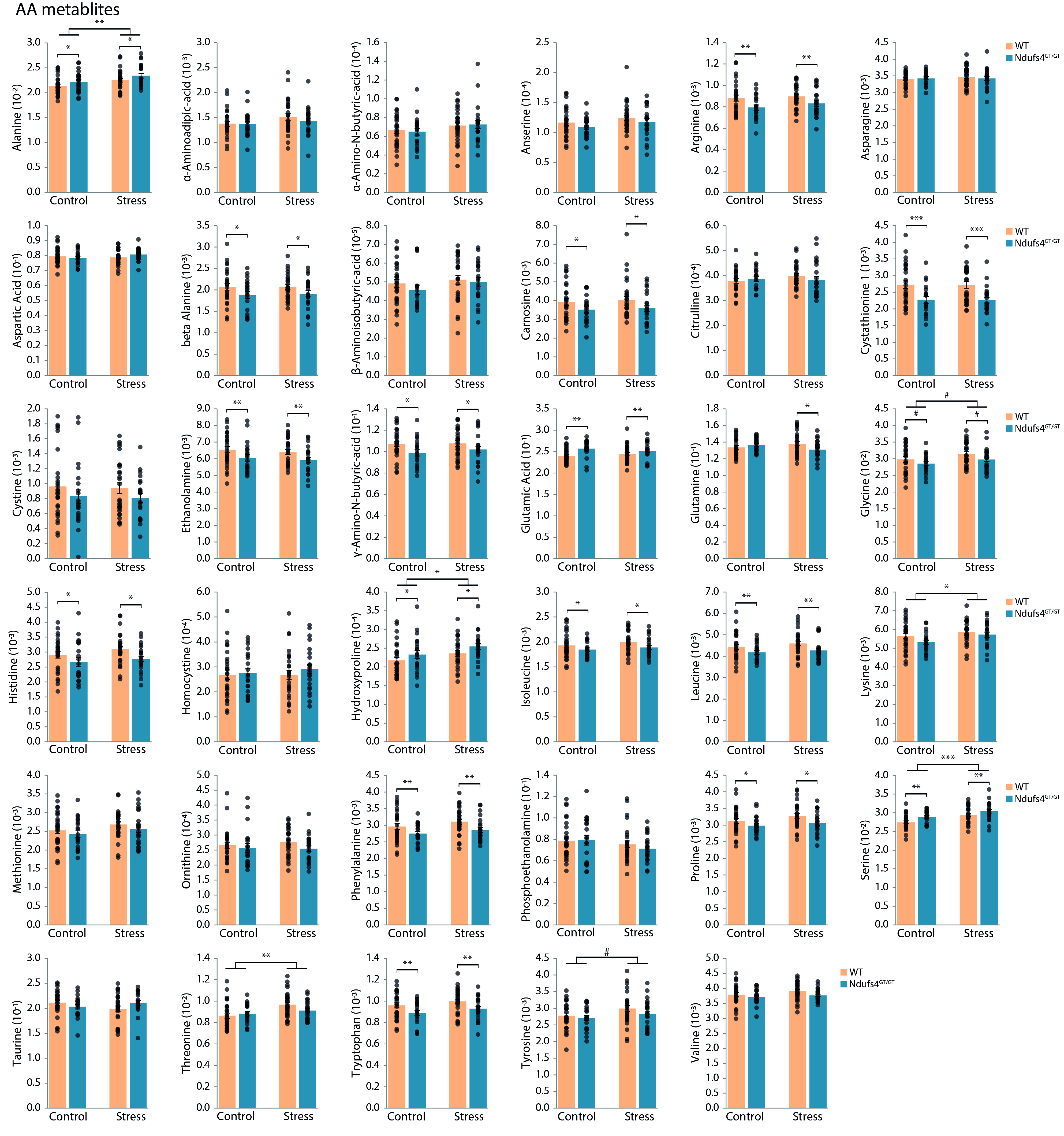

Supplement: Supplementary file 10 — Figure S9 [file 41398_2020_858_MOESM10_ESM.tif]

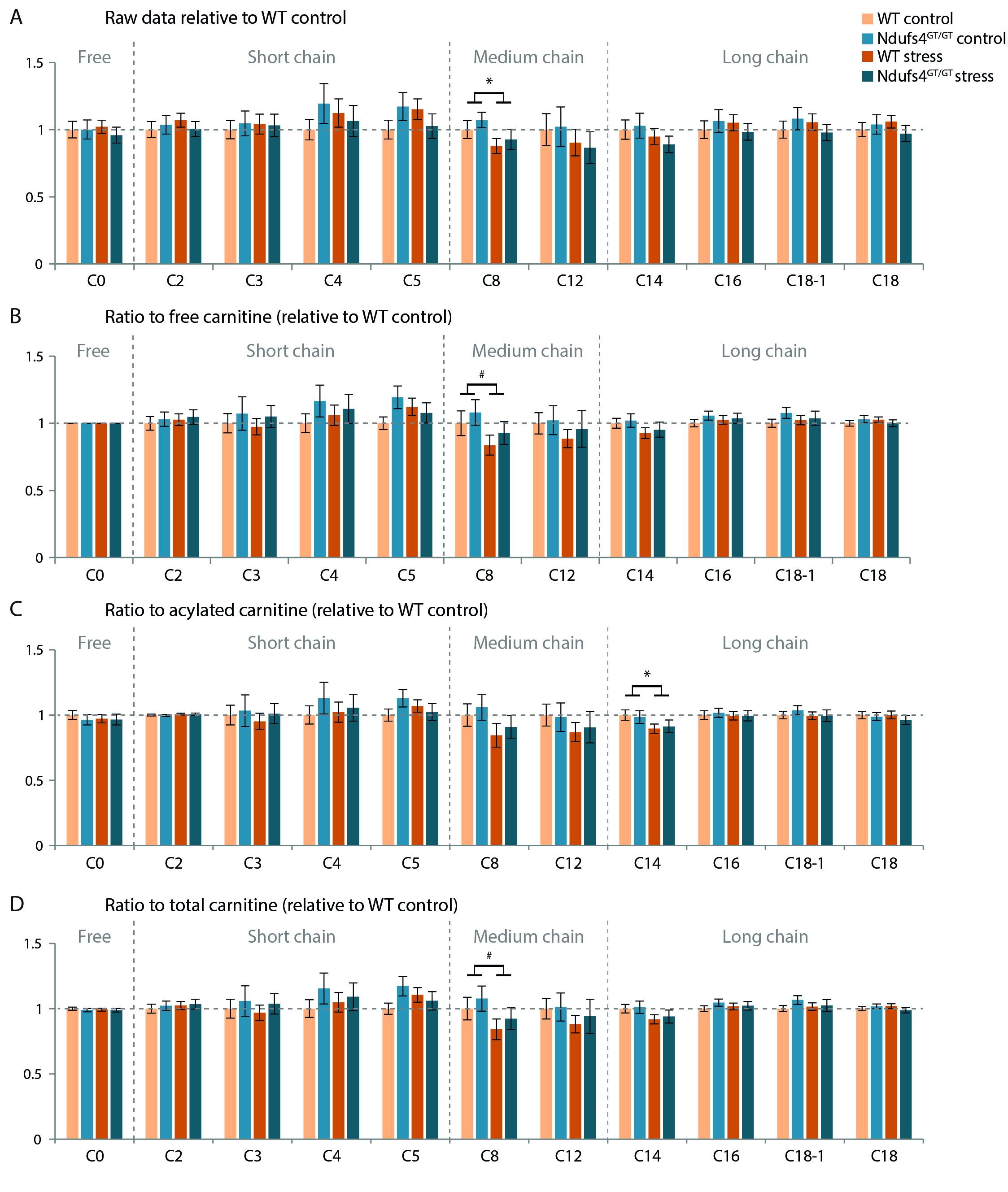

Supplement: Supplementary file 11 — Figure S10 [file 41398_2020_858_MOESM11_ESM.tif]

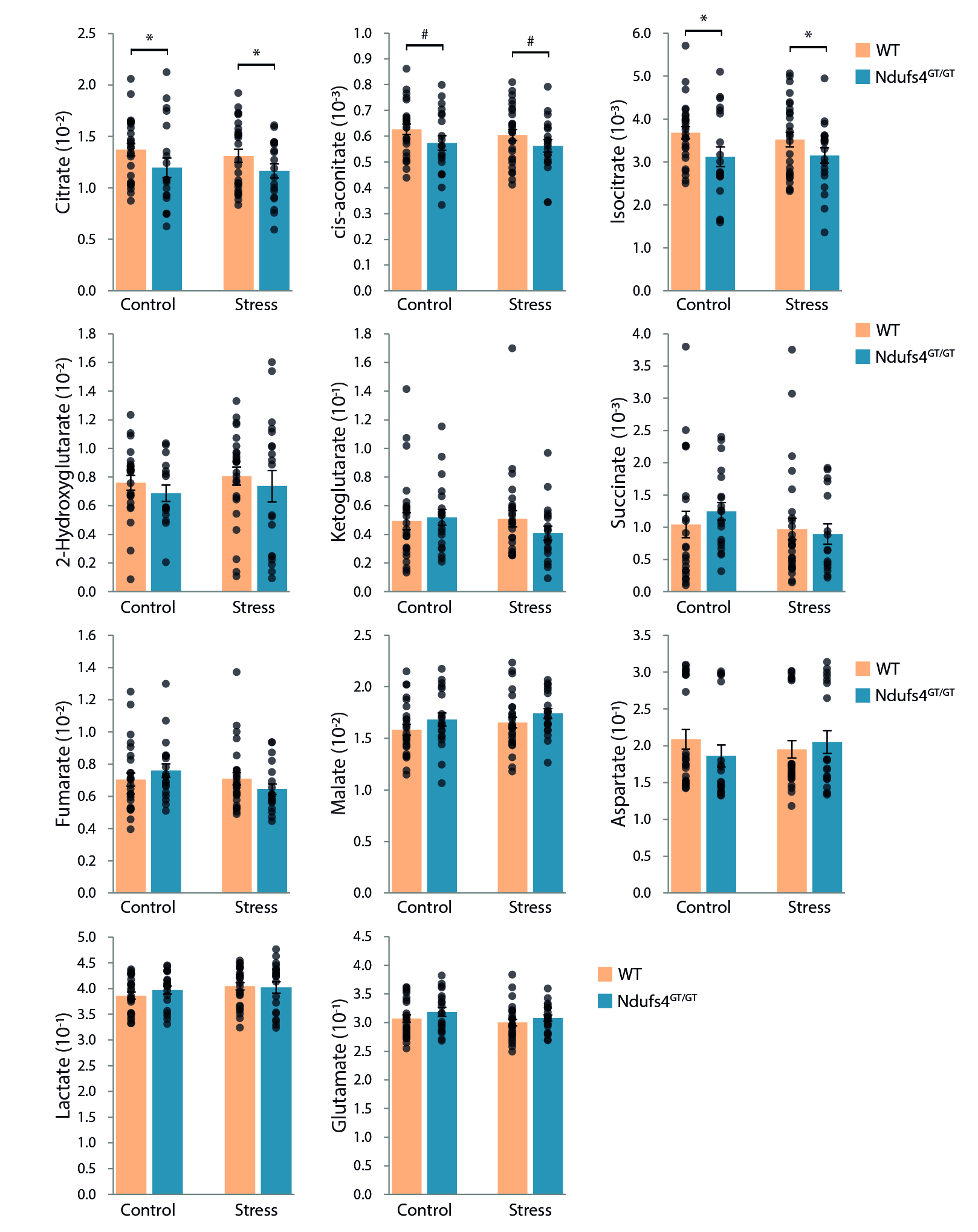

Supplement: Supplementary file 12 — Figure S11 [file 41398_2020_858_MOESM12_ESM.tif]
